# In Situ Nitric Oxide Gas Nanogenerator Reprograms Glioma Immunosuppressive Microenvironment

**DOI:** 10.1002/advs.202300679

**Published:** 2023-04-21

**Authors:** Yang Liu, Lin Cui, Xiao Wang, Weiling Miao, Yongxu Ju, Tiandong Chen, Huiting Xu, Ning Gu, Fang Yang

**Affiliations:** ^1^ State Key Laboratory of Bioelectronics Jiangsu Key Laboratory for Biomaterials and Devices School of Biological Sciences and Medical Engineering Southeast University Nanjing 210096 P. R. China

**Keywords:** autophagy inhibition, chemoimmunotherapy, glioblastoma therapy, immunogenic death, nitric oxide

## Abstract

Universal chemotherapy in glioblastoma patients causes chemoresistance and further limits immune cells by creating an immunosuppressive tumor microenvironment that are difficult to solve by single‐drug therapeutic approaches. Here, this work designs hybrid drug‐loaded nanoliposomes by co‐loading the chemotherapeutic drug temozolomide (TMZ) and nitric oxide (NO) prodrug JS‐K with sphingosine‐1‐phosphate molecules (S1P) on the surface. The S1P‐S1P receptors axis endows nanoliposomes with rapid targeting and lysosomal escaping capability. Then, fine‐tuned TMZ release and NO gas production following JS‐K release in glioma microenvironment decrease chemoresistance and increase tumor immunogenicity through inhibiting the cellular autophagy as well as inducing mitochondrial dysfunction. RNA sequencing analysis demonstrates that the NO gas generation reprograms glioma microenvironment immune and inflammation‐related pathways. The positive immune response in turn effectively activates the enhanced efficacy of chemotherapy. NO gas generated nanoliposomes thus have attractive paradigm‐shifting applications in the treatment of “cold” tumors across a range of immunosuppressive indications.

## Graphical abstract

Sphingosine‐1‐phosphate modified liposomes are designed for the delivery of the chemotherapeutic drug temozolomide and nitric oxide prodrug to glioma cells. Nitric oxide produced in tumor cells enhances tumor immunogenicity by inducing immunogenic cell death, and activates the killing effect of temozolomide by inhibiting cellular autophagy. Thus, the nanoliposomes have attractive potential to treat a range of immunologically “cold” tumors.

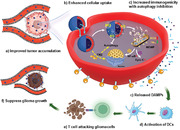

## 1 Introduction

Glioblastoma (GBM) is the most common and aggressive malignant types of central nervous system tumors.^[^ ^1^ ^]^ Although multiple approaches‐based therapeutic strategies are commonly used, including the maximal safe tumor resection, followed by chemotherapy and radiation therapy,^[^ ^2^, ^3^, ^4^, ^5^ ^]^ the therapeutic response were not satisfactory, due to the ability of residual glioma cells to exacerbate chemoresistance and promoted cell survival by enhancing their autophagy.^[^ ^6^, ^7^ ^]^

The potential of immunotherapy‐based strategies for GBM treatments has been extensively explored over the last decade.^[^ ^8^, ^9^, ^10^ ^]^ Tumor cells undergoing immunogenic cell death (ICD) can initiate an adaptive immune response and establish a long‐term immune protection, thus having the potential to enhance antitumor immunity. The emission of damage‐associated molecular patterns (DAMPs) in response to cell death is a typical immunogenic characteristic of ICD. However, glioma cells have low immunogenicity that is considered to be “cold” tumor, and enhanced autophagy further shapes the severely immunosuppressive microenvironment by degrading DAMP molecules released from cells damaged by chemotherapeutic agents, which greatly impairs the antitumor immune response.^[^ ^11^, ^12^, ^13^, ^14^ ^]^

**Figure 1:**
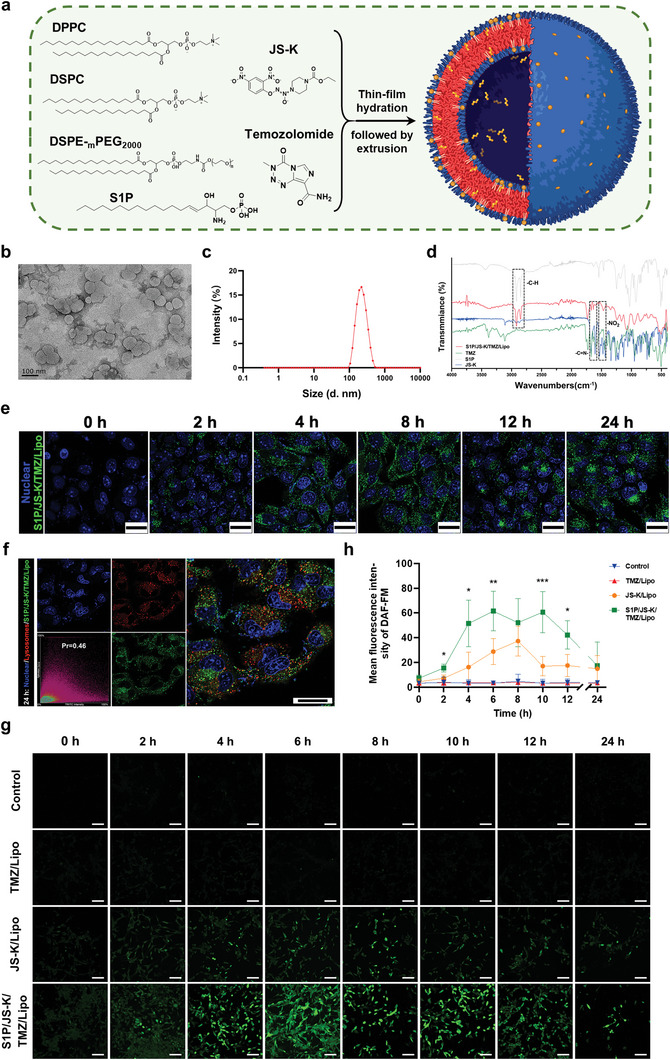
Design and characterization of S1P/JS‐K/TMZ/Lipo. a) Chemical structures of the nitric oxide (NO) prodrug O_2_‐(2,4‐Dinitrophenyl) 1‐[(4‐ethoxycarbonyl) piperazin‐1‐yl] diazen‐1‐ium‐1,2‐diolate (JS‐K), chemotherapeutic agent temozolomide (TMZ), glioma cell targeting molecule sphingosine‐1‐phosphate (S1P), 1,2‐dipalmitoyl‐sn‐glycero‐3‐phosphocholine (DPPC), 1,2‐Distearoylsn‐sn‐glycero‐3‐phosphocholine (DSPC) and 1,2‐distearoyl‐sn‐glycero‐3‐phosphoethanolamine‐N‐ [methoxy (poly (ethylene glycol))‐2000] (DSPE‐_m_PEG_2000_). The components were assembled in the S1P/JS‐K/TMZ/Lipo through film hydration and extrusion methods, to obtain the drug encapsulation. b) Morphology of S1P/JS‐K/TMZ/Lipo by transmission electron microscopy (TEM). Scale bar, 100 nm. c) Dynamic light scattering analysis of S1P/JS‐K/TMZ/Lipo size distributions. d) Fourier transform infrared (FT‐IR) spectrum of free S1P (Grey), JS‐K (Blue), TMZ (Green) and S1P/JS‐K/Lipo (Red). e) Representation of green fluorescently labeled S1P/JS‐K/TMZ/Lipo internalized by GL261 cells after co‐incubation for different time periods (0, 2, 4, 8, 12, and 24 h). The nucleus was stained by 4′,6‐diamidino‐2‐phenylindole (DAPI, blue) to be visualized by confocal microscopy. Scale bars, 25 µm. f) Co‐localization of green fluorescently labeled S1P/JS‐K/TMZ/Lipo with LysoTracker‐labeled lysosomes (Red) at the 24 h post‐treatment timepoint. Pearson coefficient (P_r_) was 0.46 calculated using ImageJ software (Version: 2.9.0). Scale bar, 10 µm. g) Fluorescence analyses of GL261 cells pre‐treated with NO‐specific green fluorescent probe DAF‐FM DA, and post‐treatment with TMZ/Lipo, JS‐K/Lipo and S1P/JS‐K/TMZ/Lipo for different time periods (0, 2, 4, 6, 8, 10, 12, and 24 h), respectively. Scale bars, 100 µm, and h) Quantitative analysis of the fluorescence intensity of GL261 cells after different treatments for different time periods (0, 2, 4, 6, 8, 10, 12, and 24 h) in (g). ^*^ *p* < 0.05, ^**^ *p* < 0.01, and ^***^ *p* < 0.001 were calculated using one‐way analysis of variance (ANOVA) followed by Student's *t*‐test (two‐tailed).

Nanomaterials combing autophagy inhibitors with ICD inducer have been used for the treatment in cancer.^[^ ^15^ ^]^ Several chemotherapeutic agents such as oxaliplatin, paclitaxel ^[^ ^16^, ^17^ ^]^ as well as critical gas molecules such as nitric oxide (NO)^[^ ^18^ ^]^ have been demonstrated to augment immunotherapy by inducing ICD.^[^ ^19^, ^20^ ^]^ However, achieving spatiotemporally consistent autophagy inhibition and immune enhancement in gliomas is difficult, and the presence of brain‐blood‐tumor‐barrier (BBTB) limits the delivery of complex combinations of drugs. But if successful, it would provide unprecedented benefits for the treatment of gliomas. We have confirmed in previous studies that by modifying sphingosine‐1‐phosphate (S1P) molecules on the surface of liposomes, the transcytosis of caveolin‐1 in vascular endothelial cells can be utilized to achieve liposome crossing of BBTB, and successfully endocytosed by tumor cells with high expression of S1P receptors on the cell membrane, and the prodrug of nitric oxide can be delivered to the GBM.^[^ ^21^ ^]^ Using this liposomal delivery platform that can successfully achieve BBTB crossing, here we report a dual drug‐loaded nanoliposomes as intracellular NO generator, in combination with chemotherapeutic agents temozolomide (TMZ), which utilizing the dual functions of NO to enhance the killing effect of TMZ through the inhibition of tumor cell autophagy on the one hand, and simultaneously reverses the tumor immunosuppressive microenvironment by inducing ICD, resulting in efficient treatment and tumor remission in orthotopic glioma‐bearing mouse models. Such nanoliposomes containing the chemotherapeutic drug temozolomide (TMZ) and NO prodrug JS‐K with S1P molecules modified on the surface. It is named as S1P/JS‐K/TMZ/Lipo as shown in **Figure** **1**a. The S1P molecules endow S1P/JS‐K/TMZ/Lipo with blood‐brain barrier crossing capability to actively target to glioma, resulting in the sequential release of TMZ and JS‐K drugs. The fine‐tuned TMZ release and NO generation following JS‐K release produce effective chemotherapy and immunotherapy for glioma treatment. The combined administration of NO prodrug and chemotherapeutic drug liposomal deliver system could be a novel and powerful materials platform to enhance cellular immunogenicity and inhibit the chemoresistance.

## 2 Results and Discussion

### 2.1 Preparation and Characterization of S1P/JS‐K/TMZ/Lipo

We prepared a S1P/JS‐K/TMZ/Lipo delivery platform composed of synthetic phospholipid materials, such as DPPC, DSPC, DSPE‐_m_PEG_2000_, the first‐line chemotherapeutic agent TMZ commonly used for glioma, and the NO prodrug JS‐K. The phospholipid components served to encapsulate TMZ in the core, and JS‐K and S1P molecules in the lipid bilayer, through a thin‐film hydration method, followed by the assembling of nanoliposome with a bilayer core‐shell structure using an extrusion method. Transmission electron microscopy (TEM) demonstrated that S1P/JS‐K/TMZ/Lipo exhibited a spherical structure with a hydrodynamic size of ≈150 nm and a polydispersity index of 0.11, indicating that S1P/JS‐K/TMZ/Lipo had good monodispersity (Figure 1b,c). The S1P/JS‐K/TMZ/Lipo maintained excellent stability in phosphate‐buffered saline solution (PBS, pH 7.4), 10% fetal bovine serum (FBS), and in a combination of PBS (pH 7.4) and 10% FBS at room temperature (Figures S1 and S2, Supporting Information). The encapsulation of S1P, JS‐K, and TMZ in S1P/JS‐K/TMZ/Lipo was confirmed by Fourier transform infrared (FT‐IR) spectrometer‐based analysis (Figure 1d). The stretch signal at 2950–2850 cm^−1^ of alkyl groups in the free S1P, the N‐O stretch signal at 1360–1290 cm^−1^ of nitro‐aromatic groups and the C‐C stretch signal at 1610–1370 cm^−1^ of the aromatic ring in JS‐K, were detected in S1P/JS‐K/TMZ/Lipo, together with the special double bond of a C‐N stretching peak at 1680–1640 cm^−1^, typical of TMZ. These results demonstrated that the S1P, JS‐K, and TMZ molecules were successfully combined and encapsulated in the lipid bilayers of S1P/JS‐K/Lipo formulations. In addition, the high‐performance liquid chromatography (HPLC) was used to measure the efficiency in drug loading of JS‐K and TMZ in S1P/JS‐K/TMZ/Lipo nanocarriers, with values of 75.13% and 52.14%, respectively.

### 2.2 S1P/JS‐K/TMZ/Lipo is Rapidly Taken up by Cells Through S1P‐Mediated Active Targeting to Generate NO at an Intracellular Level

To gain insight into the delivery efficiency of S1P/JS‐K/TMZ/Lipo, confocal laser scanning microscopy (CLSM) was used to investigate the distribution of green fluorescent molecule DiO‐labeled S1P/JS‐K/TMZ/Lipo incubated with in GL261 cells for different time periods. Compared to DiO‐Lipo, DiO‐TMZ/Lipo and DiO‐JS‐K/Lipo, the relative mean fluorescence intensity of the DiO‐S1P/Lipo and DiO‐S1P/JS‐K/TMZ/Lipo signals in GL261 cells showed a rapid increase with the increasing co‐incubation time (Figure 1e and Figure S3, Supporting Information), indicating that S1P molecules could effectively enhance the endocytosis of liposomes by GL261 cells.

To further investigate the effect of S1P molecules on intracellular transport of S1P/JS‐K/TMZ/Lipo, we analyzed the correlation between the distribution of red fluorescence‐labeled lysosomes and DiO‐labeled liposomes (Lipo, JS‐K/TMZ/Lipo, S1P/TMZ/Lipo and S1P/JS‐K/TMZ/Lipo) co‐cultured with GL261 cells for different time periods using CLSM (Figure S4, Supporting Information). With the increasing co‐incubation time, S1P/JS‐K/TMZ/Lipo effectively escaped from the lysosomes and the Pearson correlation coefficient (P_r_) value gradually decreased to 0.46 after 24 h of co‐culture with GL261 cells (Figure 1f). By contrast, the P_r_ values of GL261 cells co‐cultured with TMZ/Lipo and JS‐K/TMZ/Lipo were not lower than 0.5, indicating that S1P molecules played a critical role in the lysosomal escape of the liposomes.

To assess the intracellular production of NO by S1P/JS‐K/TMZ/Lipo, we incubated GL261 cells after different treatments with DAF‐FM DA and measured their fluorescence intensity over time (Figure 1g,h). While no fluorescence signal was detected from the untreated or TMZ/Lipo treated GL261 cells within 24 h, clearly green fluorescent signal was present in JS‐K/Lipo or S1P/JS‐K/TMZ/Lipo‐treated GL261 cells at 2 h, and the total fluorescence of S1P/JS‐K/TMZ/Lipo‐treated GL261 cells was significantly higher than that of the JS‐K/Lipo‐treated GL261 cells up to 24 h. Altogether, these findings showed S1P/JS‐K/TMZ/Lipo might have significant advantages in rapidly distributing into the GL261 cells and producing high concentrations of intracellular NO.

### 2.3 NO Produced by S1P/JS‐K/TMZ/Lipo Enhances the Chemotherapeutic Effects of TMZ by Autophagy Inhibition

To assess the ability of S1P/JS‐K/TMZ/Lipo to enhance the killing effect of the TMZ on GL261 cells by inhibiting cellular autophagy, we analyzed the viability of GL261 cells after different treatments (free JS‐K, free TMZ, JS‐K/Lipo, TMZ/Lipo, S1P/TMZ/Lipo, and S1P/JS‐K/TMZ/Lipo).

**Figure 2:**
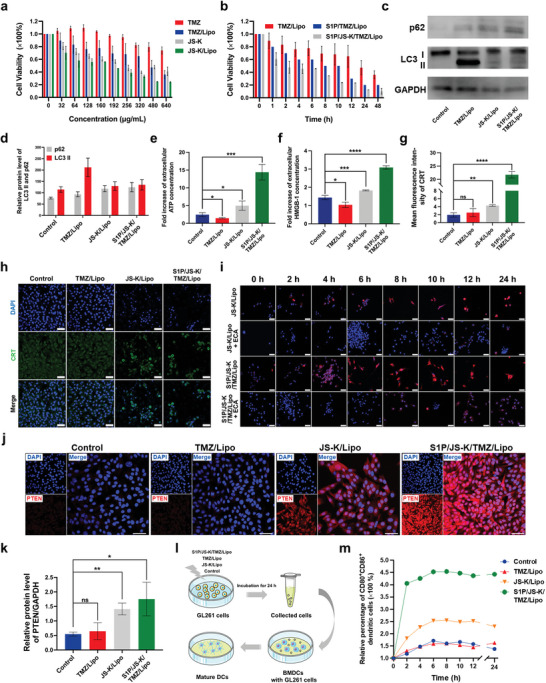
NO production induced by S1P/JS‐K/TMZ/Lipo inhibits autophagy and induces the in vitro expression of immune‐associated antigens in GL261 cells. a) Cell viability of GL261 cells after incubation with free TMZ, free JS‐K, TMZ/Lipo and JS‐K/Lipo for 24 h. The concentrations of TMZ/Lipo and JS‐K/Lipo were calculated based on the loading concentration of TMZ (0–640 *µ*g mL^−1^) and JS‐K (0–640 *µ*g mL^−1^), respectively. b) Cell viability of GL261 cells incubated with TMZ/Lipo, S1P/TMZ/Lipo and S1P/JS‐K/TMZ/Lipo at the same concentration of TMZ after different timepoints. c) Western Blot analysis for p62 and LC3 II expressions after treatment of GL261 cells with TMZ/Lipo, JS‐K/Lipo, or S1P/JS‐K/TMZ/Lipo for 24 h. d) The quantitative analysis results for p62 and LC3 II expression were calculated by normalizing p62 and LC3 II protein bands intensities at each treatment to that of glyceraldehyde‐3‐phosphate dehydrogenase (GAPDH) using ImageJ software (Version: 2.9.0). e–g) Levels of e) ATP, f) HMGB‐1, and g) CRT expression by GL261 cells after different treatments (Control, TMZ/Lipo, JS‐K/Lipo, and S1P/JS‐K/TMZ/Lipo) for 24 h. h) CLSM images of CRT in GL261 cells after incubation with different treatments (Control, TMZ/Lipo, JS‐K/Lipo, and S1P/JS‐K/TMZ/Lipo) for 24 h. CRT protein was stained by anti‐CRT antibody (green), and nuclei were stained by DAPI (blue). Scale bars, 50 µm. i) CLSM images of CRT in GL261 cells after incubation with different treatments (JS‐K/Lipo, JS‐K/Lipo with ECA, S1P/JS‐K/TMZ/Lipo, S1P/JS‐K/TMZ/Lipo with ECA) for 24 h. CRT protein was stained by anti‐CRT antibody (red), and nuclei were stained by DAPI (blue). Scale bars, 50 µm. j) CLSM images and k) mean fluorescence intensity analysis of PTEN expression in GL261 cells after different treatment (Control, TMZ/Lipo, JS‐K/Lipo, and S1P/JS‐K/TMZ/Lipo) for 24 h. Scale bars, 50 µm. l) Schematic diagram showing the process of bone marrow‐derived dendritic cells (BMDCs) maturation in 48 h in response to different pre‐treatments of GL261 cells (Control, TMZ/Lipo, JS‐K/Lipo, and S1P/JS‐K/TMZ/Lipo) for 24 h. m) Flow cytometry measurement of BMDCs activation. Data represent mean ± SD, and three independent experiments (*n* = 3) were performed. The statistical significance is indicated by ^*^ *p* < 0.05, ^**^ *p* < 0.01, ^***^ *p* < 0.001, and ^****^ *p* < 0.0001 determined using one‐way analysis of variance (ANOVA) followed by Student's *t*‐test (two‐tailed).

Both TMZ and JS‐K showed concentration‐dependent cytotoxicity. Free TMZ showed negligible toxicity against GL261 cells at concentrations below 480 µg mL^−1^, indicating that glioma cells are strongly resistant to TMZ. The IC_50_ of free JS‐K against GL261 cells was 225 µg mL^−1^ (**Figure** **2**a). By contrast, we found that liposome formulation promoted cellular uptake of the TMZ and JS‐K, and the IC_50_ of TMZ/Lipo and JS‐K/Lipo against GL261 cells were reduced to 500 and 180 µg mL^−1^, we thus fixed the loading concentrations of TMZ at 500 µg mL^−1^ and JS‐K at 180 µg mL^−1^. Benefiting from the active targeting ability of S1P, the cytotoxic effect of S1P/TMZ/Lipo on GL261 cells was increased, while S1P/JS‐K/TMZ/Lipo showed a higher cytotoxicity effect against GL261 cells than both S1P/TMZ/Lipo and TMZ/Lipo (Figure 2b), indicating that NO further enhanced the sensitivity of GL261 cells to TMZ. Meanwhile, the cell toxicity of S1P/JS‐K/TMZ/Lipo at determined drug concentrations was investigated in mouse brain capillary endothelial cell line (bEnd.3) and mouse hippocampal neurons cell line (HT22). As shown in Figure S5, Supporting Information, within the co‐incubation time range of 0–48 h, no apparent cytotoxicity was observed toward both bEnd.3 and HT22 cells.

To explore the mechanism by which NO produced by JS‐K increased the sensitivity of GL261 cells to TMZ, a western blot assay was used to analyze the expression levels of autophagy‐associated proteins (Figure 2c). Compared to untreated GL261 cells, TMZ/Lipo‐treated GL261 cells showed higher expression level of LC3 II and lower expression levels of p62, where the ratio of the relative expression levels of LC3 II to LC3 I was 2.26‐fold higher than that of the control group, demonstrating the enhancement of the autophagy within GL261 cells after the treatment with TMZ. However, compared to TMZ/Lipo, the treatment of GL261 cells with S1P/JS‐K/TMZ/Lipo reversed the cellular autophagy, with a decrease of 36.44% in LC3 II expression and an increase of 33.68% in p62 expression (Figure 2d and Figure S6, Supporting Information). Therefore, it demonstrates that the NO production is a key factor in inhibiting cellular autophagy to improve the sensitivity of GL261 cells to TMZ chemotherapy.

### 2.4 S1P/JS‐K/TMZ/Lipo‐Derived NO Production Activated Dendritic Cells with Enhanced Cellular Immunogenicity by Inducing Immunogenic Cell Death Effect

We further examined the effect of S1P/JS‐K/TMZ/Lipo on the immunogenicity of GL261 cells by analyzing the release of cellular DAMPs, including adenosine‐triphosphate (ATP), high mobility group box‐1 protein (HMGB1) and calreticulin (CRT).

The release of ATP and HMGB‐1 was reduced in TMZ/Lipo‐treated GL261 cells compared to untreated groups, proving that TMZ inhibited the release of DAMPs. By contrast, JS‐K/Lipo‐treated GL261 cells showed increased release of ATP and HMGB‐1, as well as the expression level of CRT on the cell surface (Figure 2e–h). The release of DAMPs from S1P/JS‐K/TMZ/Lipo‐treated GL261 cells was further increased compared to JS‐K/Lipo, while the expression of CRT protein in GL261 cells incubated with JS‐K/Lipo or S1P/JS‐K/TMZ/Lipo was significantly attenuated by the addition of etacrynic acid (ECA) (Figure 2i), which inhibited the GST enzyme that is essential for the production of NO by JS‐K, indicating that NO is a key factor in inducing a considerable release of DAMPs from GL261 cells.

To gain insight into the differences in cellular immunogenicity of GL261 cells after different treatments, the levels of phosphatase and tensin homolog (PTEN) expressed by GL261 were investigated (Figure 2j). There was no significant difference (*p* = 0.084) in PTEN expression in GL261 cells after TMZ/Lipo treatment compared to the untreated group, whereas a remarkable increase in PTEN expression was observed in GL261 cells after treatment with S1P/JS‐K/TMZ/Lipo (Figure 2k), demonstrating that the intracellular production of NO upregulates the PTEN expression in glioma cells and increased immunogenicity of glioma cells.

To evaluate the activation of immune‐related cells by GL261 cells after S1P/JS‐K/TMZ/Lipo treatment, we incubated mouse bone marrow‐derived dendritic cells (BMDC) with GL261 cells for 48 h and quantitatively analyzed by flow cytometry (Figure 2l). When co‐cultured with S1P/JS‐K/TMZ/Lipo‐treated GL261 cells, a significant increase in the proportion of BMDC cells with high expression of both CD80 and CD86 was observed, which were 3.22, 2, and 1.93‐fold higher than those in the control, TMZ/Lipo and JS‐K/Lipo‐treated group (Figure 2m), suggesting that GL261 cells with improved immunogenicity after S1P/JS‐K/TMZ/Lipo treatment significantly promote the BMDC maturation.

### 2.5 The Enhancement of Chemotherapy Efficacy and Immunogenicity by S1P/JS‐K/TMZ/Lipo Delivery Systems Caused Mitochondrial Dysfunction

Since emission of DAMPs is associated with activation of the stress response,^[^ ^22^ ^]^ we investigated the differences in the mitochondrial stress response of GL261 cells after different treatments.

**Figure 3:**
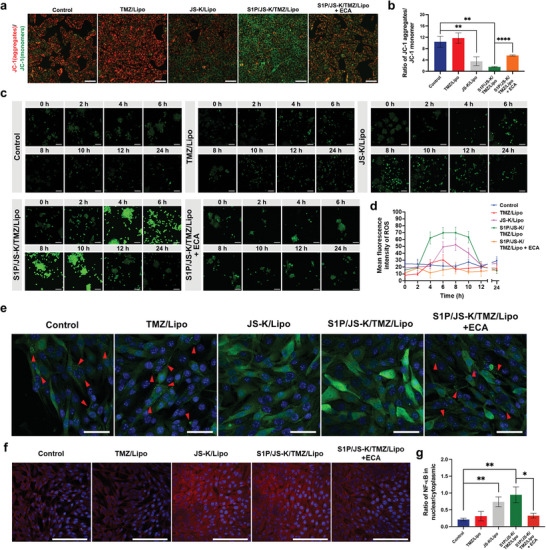
NO triggers ICD‐associated DAMPs releasing through mitochondrial dysfunction in GL261 cells. a) CLSM images of mitochondrial membrane potential (MMP) in GL261cells after treatment with TMZ/Lipo, JS‐K/Lipo, S1P/JS‐K/TMZ/Lipo, and S1P/JS‐K/TMZ/Lipo along with ECA, respectively. Scale bars, 100 µm. b) MMP of GL261 cells in a), the fluorescence intensity ratio (JC‐1 aggregates/monomer) was used to determine the extent of the MMP. c) CLSM images of the intracellular ROS levels in GL261cells after treatment with TMZ/Lipo, JS‐K/Lipo, S1P/JS‐K/TMZ/Lipo, and S1P/JS‐K/TMZ/Lipo along with ECA, respectively. Scale bars, 100 µm. d) Quantitative analysis of the mean fluorescence intensity of GL261 cells in (c). e) CLSM images of cytochrome *c* in GL261 cells after treatment with TMZ/Lipo, JS‐K/Lipo, S1P/JS‐K/TMZ/Lipo, and S1P/JS‐K/TMZ/Lipo along with ECA, respectively. Scale bars, 50 µm. f) CLSM images of NF‐*κ*B p65 in GL261 cells after treatment with TMZ/Lipo, JS‐K/Lipo, S1P/JS‐K/TMZ/Lipo, and S1P/JS‐K/TMZ/Lipo along with ECA, respectively. Scale bars, 50 µm. g) Quantitative analysis of nuclear‐cytoplasmic ratio of NF‐*κ*B p65 in GL261 cells in (f). Data represent mean ± SD, and three independent experiments (*n* = 3) were performed. The statistical significance is indicated by ^*^ *p* < 0.05, ^**^ *p* < 0.01, and ^****^ *p* < 0.0001 determined using one‐way analysis of variance (ANOVA) followed by Student's *t*‐test (two‐tailed).

JS‐K/Lipo and S1P/JS‐K/TMZ/Lipo efficiently reduced the mitochondrial membrane potential (MMP) accompanying with significantly increased intracellular levels of reactive oxygen species (ROS). By contrast, TMZ/Lipo had almost no effect on MMP and ROS in GL261 cells. However, the ability of S1P/JS‐K/TMZ/Lipo to disrupt the intracellular MMP and ROS microenvironment was significantly attenuated when GL261 cells were pre‐treated with ECA (**Figure** **3**a–d), demonstrating that intracellular NO production by S1P/JS‐K/TMZ/Lipo might trigger mitochondrial stress by regulating the MMP and ROS microenvironment, which may be a critical pathway for the induction of ICD.

Motivated by these observations, we also examined modulation of mitochondrial outer membrane permeabilization (MOMP) in GL261 cells treated by S1P/JS‐K/TMZ/Lipo. Considering the release of mitochondrial cytochrome *c* as a marker of MOMP, we examined the intracellular distribution of cytochrome *c* (Figure 3e). Cytochrome *c* was weakly distributed in untreated GL261 cells, while diffusely staining was shown in S1P/JS‐K/TMZ/Lipo‐treated GL261 cells. However, the distribution of cytochrome *c* in the GL261 cells remained dotted pattern when ECA‐pretreated GL261 cells incubated with S1P/JS‐K/TMZ/Lipo, suggesting that S1P/JS‐K/TMZ/Lipo induced the translocation of cytochrome *c* into the cytoplasm. Furthermore, S1P/JS‐K/TMZ/Lipo treatment induced translocation of nuclear factor‐kB (NF‐kB) (Figure 3f,g), a key regulator of the inflammatory response, to the GL261 cells nuclear, suggesting that engaging MOMP could trigger the release of immunogenic DAMPs.

### 2.6 RNA Sequencing for Evaluating the Effect of Combined Chemotherapy and Immunotherapy Induced by S1P/JS‐K/TMZ/Lipo

**Figure 4:**
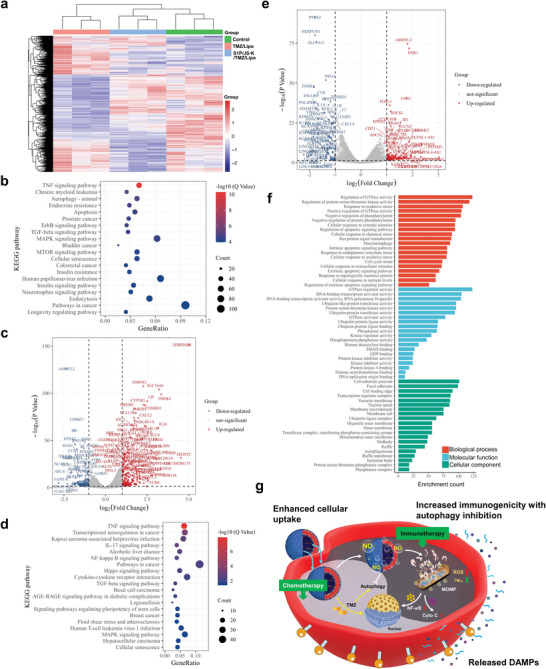
Transcriptome analysis of S1P/JS‐K/TMZ/Lipo‐treated GL261 cells. a) Heatmap representation and cluster analysis of significantly upregulated and downregulated genes in GL261 cells after treatment with TMZ/Lipo, S1P/JS‐K/TMZ/Lipo, or PBS. Three biological replicated are presented. b) Top 20 KEGG pathways in GL261 cells after TMZ/Lipo treatment. Gene ratio (*x*‐axis) is the percentage of significant genes over the total genes in a given pathway. c) Volcano plot analysis of GL261 cells after TMZ/Lipo treatment versus control. The abscissa represents the difference between the two data sets following log2 conversion and the ordinate represents the ‐log10 (*p*‐value) calculated by unpaired Student's *t*‐test. Red dots represent significantly upregulated genes, blue dots represent significantly downregulated genes. d) Top 20 KEGG pathways in GL261 cells after S1P/JS‐K/TMZ/Lipo treatment. Gene ratio (*x*‐axis) is the percentage of significant genes over the total genes in a given pathway. e) Volcano plot analysis of GL261 cells after S1P/JS‐K/TMZ/Lipo treatment versus control. The abscissa represents the difference between the two data sets following log2 conversion and the ordinate represents the ‐log10 (*p*‐value) calculated by unpaired Student's *t*‐test. Red dots represent significantly upregulated genes, blue dots represent significantly downregulated genes. f) Top 20 GO terms of three categories (biological processes, molecular functions, and cellular components) in GL261 cells after S1P/JS‐K/TMZ/Lipo treatment. Gene counts (*x*‐axis) is the number of genes present in this GO term. g) Schematic diagram of the possible mechanism for S1P/JS‐K/TMZ/Lipo to achieve the combination of chemotherapy and immunotherapy for GL261 cells.

To further study the detailed antitumor mechanism of S1P/JS‐K/TMZ/Lipo‐mediated chemoimmunotherapy, the transcriptome sequencing of GL261 cells treated by three different systems (PBS, TMZ/Lipo, and S1P/JS‐K/TMZ/Lipo) was performed. Both principal component analysis and cluster analysis showed obvious differences among S1P/JS‐K/TMZ/Lipo, TMZ/Lipo and PBS treatment groups (**Figure** **4**a, Figures S7 and S8a, Supporting Information). After the S1P/JS‐K/TMZ/Lipo treatment, 230 upregulated differential genes and 181 downregulated differential genes (DEGs) in GL261 cells were evaluated (Figure S8b, Supporting Information).

In the Kyoto Encyclopedia of Genes and Genome (KEGG) pathway analysis, TMZ/Lipo treatment significantly increased the autophagy‐related signaling pathway proteins (Figure 4b). For example, the expression of heme oxygenase‐1 (HMOX1) protein was upregulated by 2.27‐fold. Notably, the TMZ/Lipo treatment group showed a significant upregulation of proteins that play a negative effect on immune regulatory pathways, such as inhibin subunit Beta A (INHBA), gremlin‐1 (GREM1), and dual specificity phosphatase 1 (DUSP1).^[^ ^23^, ^24^, ^25^ ^]^ They were upregulated by 3.35, 1.75, and 1.10‐fold, respectively (Figure 4c). By contrast, S1P/JS‐K/TMZ/Lipo treatment significantly enhanced several signaling pathways closely associated with improved immunogenicity of GL261 glioma cells, including TNF signaling pathway,^[^ ^26^ ^]^ NF‐kB signaling pathway,^[^ ^27^ ^]^ and MAPK signaling pathway^[^ ^28^ ^]^ (Figure 4d). For example, elevated expression of CCN1 (Cellular Communication Network Factor 1) was able to regulate immune cell transport by attracting and locally immobilizing immune cells,^[^ ^29^ ^]^ and angiomotin‑like 2 (AMOTL‐2) could inhibit glioma proliferation, migration and invasion by regulating *β*‐catenin nuclear translocation.^[^ ^30^ ^]^ Notably, the expression of HMOX1 in the autophagy‐related signaling pathway was suppressed in the S1P/JS‐K/TMZ/Lipo treatment group, suggesting that the combination of chemotherapeutic agents with NO delivery can enhance the immune inflammatory response of glioma cells by inhibiting the autophagy signaling pathway (Figure 4e).

Based on the gene ontology (GO) analysis on differential genes in biological processes, it was observed that genes that responded to stress, signal transduction, and immune system were more upregulated in the S1P/JS‐K/TMZ/Lipo group (Figure 4f). These results confirmed that the activation of the immune system in our work was by stimulating stress first, then signal transduction, and finally inducing immunity. Furthermore, the enrichment of cellular component and molecular function by GO analysis was mostly associated with immunogenicity‐related complexes, such as organelle outer membranes, mitochondrial outer membranes and autophagosomes. This observation suggested that a more robust immune response might be triggered after S1P/JS‐K/TMZ/Lipo treatment.

Thus, a schematic diagram of the possible mechanism for S1P/JS‐K/TMZ/Lipo to achieve the combination of chemotherapy and immunotherapy for GL261 cells was presented in Figure 4g. S1P/JS‐K/TMZ/Lipo produced NO by reaction with GSH catalyzed by GST in the cytoplasm of glioma cells after achieving lysosomal escape due to S1P‐S1PR axis modulation. NO inhibited cellular autophagy to enhance the chemotherapeutic killing ability of TMZ on glioma cells. Meanwhile, it promoted the loss of MMP and production of ROS in glioma cells, triggering mitochondrial stress and MOMP activation, leading to massive release of DAMP and inducing ICD of glioma cells.

### 2.7 Biodistribution of S1P/JS‐K/TMZ/Lipo in Orthotopic Glioma‐Bearing Models

**Figure 5:**
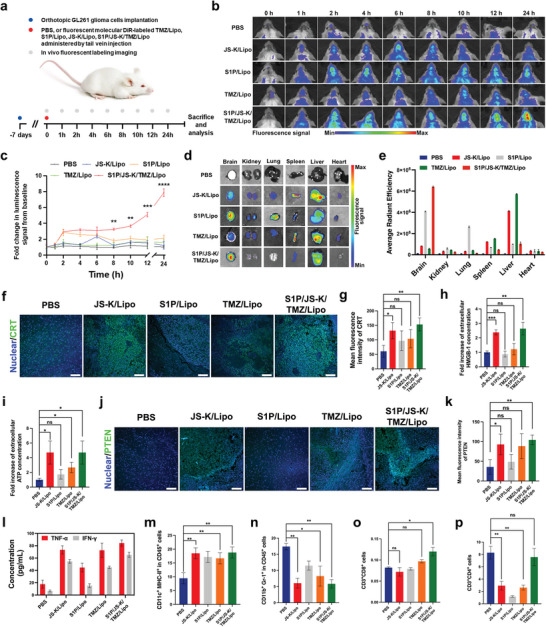
S1P/JS‐K/TMZ/Lipo elicits an antitumor immune response in in situ glioma cells. a) Experimental timeline of tumor inoculation and in vivo real‐time NIR fluorescence imaging to observe the distribution of different treatments in GL261 orthotopic glioma–bearing BALB/c mice. 7 days after glioma inoculation, mice were treated with PBS, S1P/Lipo, JS‐K/Lipo, TMZ/Lipo, or S1P/JS‐K/TMZ/Lipo by tail vein injection (JS‐K dose, 2.5 mg kg^−1^; TMZ dose, 5 mg kg^−1^). b) In vivo real‐time NIR fluorescence images of the GL261 glioma‐bearing mice at different post‐ injection time points (0, 1, 2, 4, 6, 8, 10, 12, and 24 h) of different treatments (PBS, S1P/Lipo, TMZ/Lipo, JS‐K/Lipo, and S1P/JS‐K/TMZ/Lipo). c) The mean fluorescence intensity obtained from the brain regions of mice described in (b). d) In vivo real‐time NIR fluorescence images of major organs (heart, liver, spleen, lung, and kidney) and brain tumors collected from glioma‐bearing mice at 24 h post‐injection with different treatments (PBS, S1P/Lipo, TMZ/Lipo, JS‐K/Lipo, and S1P/JS‐K/TMZ/Lipo). e) The mean fluorescence intensity of major organs (heart, liver, spleen, lung, and kidney) and brain tumors collected from glioma‐bearing mice described in (d). f) Representative CLSM images of the CRT (Green) in brain sections of GL261 glioma‐bearing mice after 24 h with different treatments (PBS, S1P/Lipo, TMZ/Lipo, JS‐K/Lipo, and S1P/JS‐K/TMZ/Lipo). Scale bars, 100 µm. g) The mean fluorescence intensity of CRT in brain sections of mice described in (f). h) Quantification the levels of HMGB‐1and i) ATP from brain tissues of GL261 glioma‐bearing mice described in (f). j) Representative CLSM images of the PTEN (Green) in brain sections of GL261 glioma‐bearing mice after 24 h with different treatments (PBS, S1P/Lipo, TMZ/Lipo, JS‐K/Lipo, and S1P/JS‐K/TMZ/Lipo). Scale bars, 100 µm. k) The mean fluorescence intensity of PTEN in brain sections of mice described in (j). l) Quantification of proinflammatory cytokines, including IFN‐g and TNF‐a, in the brain glioma tissues of GL261 glioma‐bearing mice after 24 h post‐injection with different treatments (PBS, S1P/Lipo, TMZ/Lipo, JS‐K/Lipo, and S1P/JS‐K/TMZ/Lipo). The proportions of m) CD11c^+^MHC‐II^+^ dendritic cells, n) CD11c^+^Gr‐1^+^ dendritic cells, o) CD3^+^CD8^+^ T killer cells and p) CD3^+^CD4^+^ T helper cells in the brain tissues of GL261 glioma‐bearing mice after 24 h post‐injection with different treatments (PBS, S1P/Lipo, TMZ/Lipo, JS‐K/Lipo, and S1P/JS‐K/TMZ/Lipo) were determined by flow cytometry. Data represent mean ± SD, and five independent experiments (*n* = 8) were performed. The statistical significance is indicated by ^*^ *p* < 0.05, ^**^ *p* < 0.01, ^***^ *p* < 0.001, and NS > 0.05 determined using one‐way analysis of variance (ANOVA) followed by Student's *t*‐test (two‐tailed).

We measured the biodistribution profiles of DiR‐labeled JS‐K/Lipo, S1P/Lipo, TMZ/Lipo, and S1P/JS‐K/TMZ/Lipo in orthotopic glioma‐bearing models following intravenous administration (**Figure** **5**a and Figure S9, Supporting Information). DiR‐TMZ/Lipo accumulated slowly in the brain glioma region with a weak increase 10 h post‐administration, DiR‐JS‐K/Lipo accelerated to 6 h, whereas DiR‐S1P/JS‐K/TMZ/Lipo displayed a repaid targeting ability to the brain tumor region within 2 h. The accumulated fluorescence intensity was 2.41 and 2.02‐fold higher than those in the DiR‐TMZ/Lipo and DiR‐JS‐K/Lipo group, respectively (Figure 5b,c). Thus, S1P/JS‐K/TMZ/Lipo exhibited greater rapid targeting of brain glioma region than TMZ/Lipo or JS‐K/Lipo.

Biodistribution analysis at 24 h in animals bearing orthotopic established GL261 glioma revealed considerably higher glioma accumulation of S1P/JS‐K/TMZ/Lipo, which was 55.26, 7.74, 11.32, and 1.57‐fold of that in the brain of the control, JS‐K/Lipo, TMZ/Lipo, and S1P/Lipo (Figure 5d). S1P/JS‐K/TMZ/Lipo also accumulated in spleen and liver, but exhibited low uptake in other tissues. As expected, the spleen and liver were abundant sites of uptake for both TMZ/Lipo and JS‐K/Lipo (Figure 5e). The fluorescence intensity ratio in the brain and the liver (B/L) was used as an index to evaluate the selective accumulation ability of S1P/JS‐K/TMZ/Lipo (Figure S10, Supporting Information), the B/L ratio of S1P/JS‐K/TMZ/Lipo was 6.10, which was substantially higher than those obtained in the PBS (1.40), JS‐K/Lipo (0.13), S1P/Lipo (4.12), and TMZ/Lipo (0.10) groups. Thus, S1P/JS‐K/TMZ/Lipo exhibited greater total accumulation in brain glioma region.

### 2.8 Reprogramming of Glioma Immunosuppressive Microenvironment by S1P/JS‐K/TMZ/Lipo

In preliminary therapeutic studies, mice bearing orthotopic GL261 tumors were treated with a single dose of S1P/JS‐K/TMZ/Lipo (TMZ: 5 mg kg^−1^, JS‐K: 2.5 mg kg^−1^), the equivalent dose of JS‐K/Lipo and TMZ/Lipo. As shown in Figure 5f, TMZ/Lipo treatment had no effect on CRT green fluorescence intensity in the brain tumor region, whereas the CRT green fluorescence in the brain tumor region was significantly increased in the S1P/JS‐K/TMZ/Lipo treatment groups, which was 2.17 and 2.57‐fold higher than that in TMZ/Lipo and JS‐K/Lipo treatment groups (Figure 5g). A significantly higher expression of HMGB‐1 protein (Figure 5h) and ATP (Figure 5i) in brain tumor tissues in the S1P/JS‐K/TMZ/Lipo‐treated group. Subsequently, an immunofluorescence staining for PTEN on brain tumor sections was performed to confirm the increased immunogenicity. As shown in Figure 5j, the high expression region of PTEN in the S1P/JS‐K/TMZ/Lipo treatment group highly overlapped with the glioma region, and the expression of PTEN was significantly increased (Figure 5k).

The release of DAMPs triggered by S1P/JS‐K/TMZ/Lipo indicated reprogram of the glioma immunosuppressive microenvironment. By 24 h S1P/JS‐K/TMZ/Lipo treatment, there was a trend toward increased pro‐inflammatory cytokines (TNF‐a and IFN‐g) in tumor tissue (Figure 5l). We postulated that S1P/JS‐K/TMZ/Lipo treatment ensures that dendritic cells engulfing glioma cells and tumor‐killing T cells become fully activated by virtue of the advantage of an adequate enhancement of the immunogenicity of glioma cells (Figure S11, Supporting Information). Following treatment with JS‐K/Lipo, TMZ/Lipo or S1P/JS‐K/TMZ/Lipo, the proportion of mature dendritic cells (CD11c^+^MHC‐II^+^) was significantly increased in glioma tissues (Figure 5m). Meanwhile, the proportion of myeloid‐derived suppressor cells (CD11b^+^Gr‐1^+^) was further attenuated in S1P/JS‐K/TMZ/Lipo compared to the TMZ/Lipo (Figure 5n), indicating that S1P/JS‐K/TMZ/Lipo enhanced the ability of dendritic cells to present antigens from immunogenic dead GL261 cells. As shown in Figure 5o and Figure S12, Supporting Information, the proportion of CD8^+^ T cells in brain tissue of mice treated with S1P/JS‐K/TMZ/Lipo was significantly increased, which was 1.46, 1.52, 1.24, and 1.67‐folds higher than that in the PBS, TMZ/Lipo, S1P/Lipo and JS‐K/Lipo treatment groups, respectively. Meanwhile, the proportion of CD4^+^ T cells in the brain tissue of S1P/JS‐K/TMZ/Lipo‐treated mice showed a significant decrease compared to that in the PBS‐treated group (Figure 5p). The proportion of CD8^+^ T cells in the PBS‐treated group was only one percent of CD4^+^ T cells, whereas the ratio of CD8^+^ to CD4^+^ cells in the brain tissues of the S1P/JS‐K/TMZ/Lipo‐treated group was increased to 1.6‐folds that of the PBS‐treated group. These results demonstrated that S1P/JS‐K/TMZ/Lipo stimulated antigen‐presenting dendritic cells maturation and subsequent infiltration from cytotoxic T lymphocytes.

### 2.9 Therapeutic Efficacy and Safety of S1P/JS‐K/TMZ/Lipo

**Figure 6:**
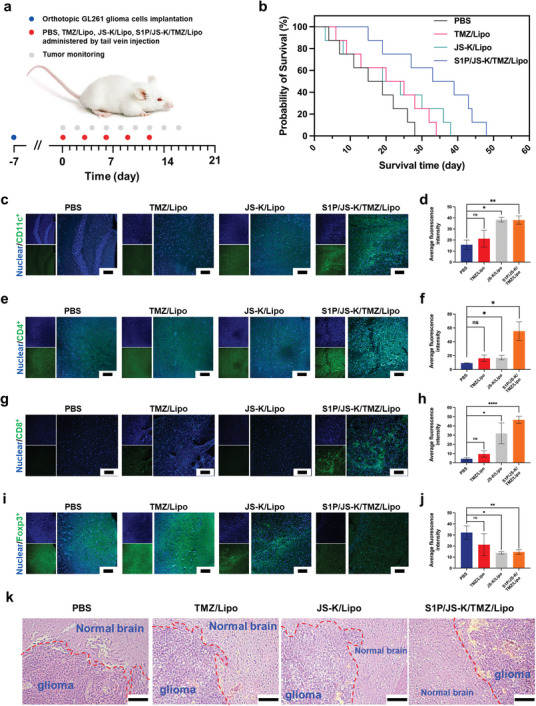
S1P/JS‐K/TMZ/Lipo promoted an enhancement in the therapeutic effect for in situ GL261 by the combined use of chemotherapy and immunotherapy. a) Treatments approaches for in situ glioma tumor (PBS, TMZ/Lipo, JS‐K/Lipo, and S1P/JS‐K/TMZ/Lipo). The GL261 glioma‐bearing mice were treated every 3 days for a total of five doses. b) Kaplan‐Meier survival curve of mice treated with schedule described in (a). c) Representative immunofluorescence images of CD11c^+^ dendritic cells in brain tissues after the last treatment. Scale bars, 100 µm. d) Mean fluorescence intensity values of CD11c^+^ dendritic cells within tumor regions from mice described in (c). e) Representative immunofluorescence images of CD4^+^ T cells in brain tissues after the last treatment. Scale bars, 100 µm. f) Mean fluorescence intensity values of CD4^+^ T cells in tumor regions from mice described in (e). g) Representative immunofluorescence images of CD8^+^ T cells in brain tissues after the last treatment. Scale bars, 100 µm. h) Mean fluorescence intensity values of CD8^+^ T cells in tumor regions from mice described in (g). i) Representative immunofluorescence images of Foxp3^+^ T cells in brain tissues after the last treatment. Scale bars, 100 µm. j) Mean fluorescence intensity values of Foxp3^+^ T cells in tumor regions from mice described in (i). k) Representative images of H&E staining of the brain section of mice after the last treatment. Scale bars, 100 µm. Data represent mean ± SD, and five independent experiments (*n* = 5) were performed. The statistical significance is indicated by ^*^ *p* < 0.05, ^**^ *p* < 0.01, ^****^ *p* < 0.0001, and NS > 0.05 determined using one‐way analysis of variance (ANOVA) followed by Student's *t*‐test (two‐tailed).

Based on the focused accumulation in brain glioma region and excellent ability to reverse the immunosuppressive microenvironment, we further evaluated that the therapeutic efficacy of S1P/JS‐K/TMZ/Lipo for glioma (**Figure** **6**a).

Compared to the median survival rate of 15 days in the PBS‐treated mice, the median survival of JS‐K/Lipo‐treated and TMZ/Lipo‐treated mice were improved by 26.7% and 33.3% at 19‐ and 20‐day post‐injection (Figure 6b), respectively. In contrast, significant inhibition of glioma growth was observed in the S1P/JS‐K/Lipo‐treated group, with a 120% increase in median survival time to 33 days, compared to the PBS‐treated group, further proving the synergistic effect of combined chemotherapy and immunotherapy in the glioma treatment.

Next, we evaluated the infiltration of immune cells in the brain glioma regions after the final dose. As shown in Figure 6c, the infiltration of CD11c^+^ dendritic cells were significantly increased in the brain glioma regions of S1P/JS‐K/TMZ/Lipo‐treated mice compared to TMZ/Lipo and JS‐K/Lipo (Figure 6d). Meanwhile, we observed that S1P/JS‐K/TMZ/Lipo significantly enhanced the infiltration of CD4^+^ T cells in GL261 tumors with an evaluated fluorescence intensity that was 5.99, 3.42, and 3.23‐folds higher than those of the PBS, TMZ/Lipo, and JS‐K/Lipo‐treated groups, respectively (Figure 6e,f).

T‐killer cells play a key role in immunotherapy to inhibit glioma growth. A significant increase of infiltration of CD8^+^ T cytotoxic cells in the GL261 tumor region was found in the S1P/JS‐K/TMZ/Lipo treatment group (Figure 6g), which was 10.69, 4.89 and 1.46‐fold higher than in the PBS, TMZ/Lipo or JS‐K/Lipo treatment groups, respectively (Figure 6h). Meanwhile, the staining of immunosuppressive Foxp3^+^ T regulatory cells in glioma tissue sections revealed that Foxp3^+^ T cells were widely distributed in the brain region in the PBS‐treated group, and TMZ/Lipo treatment further increased their distribution in glioma regions (Figure 6i), suggesting that TMZ limited the killing effect of immune cells on glioma. However, S1P/JS‐K/TMZ/Lipo treatment impaired the broad distribution of Foxp3^+^ T cells in the tumors (Figure 6j). The immune cells that abundantly accumulated in the glioma region could further promote the anti‐tumor response by secreting specific cytokines such as IFN‐g and TNF‐a. Therefore, the levels of immune‐related cytokines were measured from the serum of glioma‐affected mice using ELISA kits after the final treatment. As shown in Figure S13, Supporting Information, S1P/JS‐K/TMZ/Lipo treatment significantly increased the production of IFN‐g and TNF‐a, which were 7.75 and 10.03‐fold higher than those induced in the PBS group, respectively.

To further confirm the enhanced anti‐tumor efficacy, the dissected tumors from different treatments were stained by hematoxylin and eosin (H&E) to determine the destruction of the tumor cells. Notably, the S1P/JS‐K/TMZ/Lipo‐treated mice showed significant internal necrosis in the glioma region with well‐defined rounded edges, and no local infiltration of glioma in the normal brain parenchyma was observed. However, mice treated with PBS, TMZ/Lipo, or JS‐K/Lipo had irregularly shaped gliomas with distinct edges of glioma infiltration (Figure 6k). These differences proved the ability of S1P/JS‐K/TMZ/Lipo in inhibiting the aggressiveness of glioma tumors with a higher therapeutic efficacy.

Furthermore, the body weight of the mice treated with S1P/JS‐K/TMZ/Lipo remained largely stable during the treatment period compared to the PBS‐treated mice (Figure S14, Supporting Information). To evaluate the potential toxicity of our S1P/JS‐K/TMZ/Lipo, we carried out histology examination for healthy BALB/c mice after tail vein injection of different treatments (PBS, TMZ/Lipo, JS‐K/Lipo and S1P/JS‐K/TMZ/Lipo). On day 14, major organs (heart, liver, spleen, lung, kidney) from the treated mice were harvested and sectioned for H&E staining. No significant pathological changes were found in the major organs of S1P/JS‐K/TMZ/Lipo‐treated mice compared to PBS‐treated mice (Figure S15, Supporting Information), confirming the safety of the S1P/JS‐K/TMZ/Lipo treatment.

Taken together, these results further suggested that S1P/JS‐K/TMZ/Lipo‐based treatment promoted the formation of a new therapeutic approach to synergistically inhibit glioma growth through the combined use of the chemotherapeutic agent TMZ and the immunotherapy‐inducing agent JS‐K.

## 3 Conclusion 

It is well known that there are many challenges regarding the efficacy of the treatment for GBM, including the resistance that can be developed to chemotherapy, as well as the systemic immunosuppression following the administration of chemotherapeutic agents. Immunotherapies based on specific neoantigens, and tumor markers are currently being under evaluation in order to address these limitations. However, a reversal of the immunosuppressive effects of chemotherapeutic agents with adjuvant immune boosters may provide another treatment approach that can be translated into a viable treatment.

In the current study, we developed a nanoliposome, named S1P/JS‐K/TMZ/Lipo, which acts as an intracellular NO generator to be used in combination with the chemotherapeutic agent TMZ. This nanoliposome not only enabled the immunogenicity enhancement of glioma cells via NO production but also inhibited autophagy of glioma cells, which led to an increasing of the chemotherapy efficacy. Our in vitro studies confirmed that the NO, which was produced by S1P/JS‐K/TMZ/Lipo within the glioma cytoplasm after the lysosomal escape, was able to inhibit the cellular autophagy, improving the sensitivity of glioma cells to TMZ chemotherapy and further amplifying the effect of NO by inducing the development of mitochondrial dysfunction, which led to ICD through enhanced release of DAMPs. Additionally, our in vivo studies in orthotopic glioma‐bearing mice models demonstrated that the administration of S1P/JS‐K/TMZ/Lipo improved the immune response of the brain glioma, including an increase in DAMPs released from tumor cells and an increase in infiltration of mature dendritic cells (CD11c^+^MHC‐II^+^) and cytotoxic T cells (CD3^+^CD8^+^), accompanied by an improved survival rate of S1P/JS‐K/TMZ/Lipo‐treated animals compared to PBS‐treated or control nanoliposome–treated animals.

Our study of the therapeutic mechanisms and effects of S1P/JS‐K/TMZ/Lipo has some limitations that should be addressed. First, although S1P/JS‐K/TMZ/Lipo avoided the degradation of JS‐K before the generation of NO through lysosomal escape, the mechanism by which S1P molecules facilitated the escape of S1P/JS‐K/TMZ/Lipo from lysosomes is still unclear and requires further investigation. Furthermore, despite the apparent alteration of the immune microenvironment of glioma by the NO production, the relationship between concentrations of NO production with immune modulation effect are needed to be investigated in detail.

In conclusion, this therapeutic combination strategy through the use of liposomes provides an excellent synergistic effect, which can be applied in the future in clinical settings for the treatment of GBM and several others life‐threatening cold tumors.

## 4 Experimental Section

### Chemical and Materials

O_2_‐(2,4‐Dinitrophenyl) 1‐[(4‐ethoxycarbonyl) piperazin‐1‐yl] diazen‐1‐ium‐1,2‐diolate (JS‐K, molecular weight (M_w_ = 384.30)), (2*S*,3*R*,4*E*)‐2‐Amino‐4‐octadecene‐1,3‐diol 1‐phosphate, D‐erythro‐Sphingosine 1‐phosphate (Sphingosine‐1‐phosphate, S1P), 2‐[2,3‐Dichloro‐4‐(2‐methylene‐1‐oxobutyl)phenoxy]acetic acid (Ethacrynic acid, ECA) and 4‐methyl‐5‐oxo‐2,3,4,6,8‐pentazabicyclo [4.3.0] nona‐2,7,9‐triene‐9‐carboxamide (Temozolomide, TMZ) were all purchased from Sigma‐Aldrich (USA). 1,2‐dipalmitoyl‐*sn*‐glycero‐3‐phosphocholine (DPPC), 1,2‐Distearoylsn‐*sn*‐glycero‐3‐phosphocholine (DSPC), 1,2‐distearoyl‐*sn*‐glycero‐3‐phosphoethanolamine‐N‐[methoxy(poly(ethylene glycol))‐2000] (DSPE‐_m_PEG_2000_) were all purchased from Jiangsu Southeast Nanomaterials Co., Ltd (Huaian, China). 3,3′‐Dioctadecyloxacarbocyanine perchlorate (DiO), 1,1′‐dioctadecyl‐3,3,3′,3′‐tetramethylindotricarbocyanine iodide (DiR), and 1,1′‐Dioctadecyl‐3,3,3′,3′‐tetramethylindocarbocyanine perchlorate (DiI) were purchased from Beyotime (Haimen, China). All chemicals were analytical grade and used without further purification.

### Biological Reagents

Dulbecco's modified eagle medium (DMEM), penicillin‐streptomycin (10 000 U mL^−1^), phosphate‐buffered saline pH 7.4 (PBS), Trypsin‐EDTA (0.25%), and fetal bovine serum (FBS) were purchased from Gibco (USA). LysoTracker Red, reactive oxygen species (ROS) assay kit (DCFH‐DA), mitochondrial membrane potential detection Kit, 4′,6‐diamidino‐2‐phenylindole (DAPI), and diaminofluorescein FM diacetate (DAF‐FM DA) were purchased from Beyotime (Haimen, China). Primary antibodies and fluorescently labeled secondary antibodies used in this work included the following: Alexa Fluor 647‐labeled Goat IgG (H+L) (Abcam; catalogue no: ab150115), Alexa Fluor 555‐labeled Goat IgG(H+L) (Abcam; catalogue no: ab150078), anti p62 antibody (Abcam; catalogue no: ab109012), anti PTEN antibody (Abcam; catalogue no: ab32199), anti‐Calreticulin antibody (Abcam; catalogue no: ab22683), anti GAPDH antibody (Abcam; catalogue no: ab9485), anti CD8 antibody (Abcam; catalogue no: ab217344), anti CD4 antibody (Abcam; catalogue no: 183685), anti Foxp3 antibody (Abcam; catalogue no: ab20034), anti CD11c antibody (Abcam; catalogue no: ab52632), anti LC3B antibody (Abcam; catalogue no: ab192890). Cytokines and chemokines including tumor necrosis factor (TNF)‐*α* and TNF‐*β*, interferon (IFN)‐*γ* enzyme‐linked immunosorbent assay (ELISA) kits were purchased from BioLegend (California, USA). Other reagents and materials were purchased from Sigma‐Aldrich unless otherwise noted.

### Preparation of S1P/JS‐K/TMZ/Lipo

S1P/JS‐K/TMZ/Lipo were produced via the thin‐film hydration and membrane‐filtering extrusion method based on the previous method. Briefly, for blank Lipo (without drug or dye loading), a mixture of DPPC/DSPC/DSPE‐_m_PEG_2000_ (90/5/5 in molar ratio) in CHCl_3_ was rotary evaporated to form a thin film. Any residual organic solvent was removed by overnight evaporation under vacuum. The dried lipid film was subsequently hydrated with PBS at 60 °C for 45 min, and the lipid dispersion was extruded through polycarbonate membranes with the pore size ranging from 400 to 100 nm.

JS‐K and TMZ loaded liposomes were prepared using a traditional passive loading method. Briefly, a mixture of DPPC/DSPC/DSPE‐_m_PEG_2000_/JS‐K/S1P/TMZ in CHCl_3_ was rotary evaporated to form a thin film. The subsequent protocol was similar to blank liposomes. After extrusion, free JS‐K, S1P and TMZ were removed by G50 column.

### Liposome Structure Characterization

The transmission electron microscopy (TEM) measurements were carried out on a JEOL‐JEM 2100 electron microscope (Japan) operated at an acceleration voltage of 200 kV. In brief, liposome samples (10 µL) were dropped onto the carbon‐coated side of the 300‐mesh copper grids. After draining via a filter paper for 30 min, a phosphotungstic acid stain solution (1.5% by weight, adjusted to pH 7.0) was applied for 10 min to be imaged by TEM. UV–vis absorption, fluorescence, and luminescence spectra were acquired by either a TU‐1901 dual‐beam UV–vis spectrometer (Japan) using fused quartz cuvettes or a Tecan Infinite 200 PRO spectrometer for 96 well plates. Fourier‐transform infrared (FT‐IR) spectra were tested on an IRAffinity‐1 infrared spectrophotometer (Shimadzu, Japan) using the KBr pellet technique. Dynamic laser light scattering (DLS) measurements were performed in PBS (pH 7.4) or PBS (pH 7.4) in 10% FBS at 25 °C using a Malvern Zetasizer Nano ZS90 light scattering spectrophotometer (Malvern, British). Data were analyzed using Malvern Dispersion Technology 4.20.

### Quantitative Measurement of JS‐K and TMZ Loaded in S1P/JS‐K/TMZ/Lipo

The content of JS‐K and TMZ in S1P/JS‐K/TMZ/Lipo was determined using high performance liquid chromatography (HPLC) method. Briefly, freshly prepared liposomes were mixed with methanol solution (1:9 v/v) and then vortexed for 5 min to fully destroy the liposome structure. The JS‐K and TMZ were released from the liposomes. After filtration via a 200 nm membrane, the solution (20 µL) was measured by an HPLC system with a Phenomenex Luna 5 C18 column (Torrance, CA). The mobile phase was methanol‐acetic acid 0.5% (30:70, v/v) at a flow rate of 1 mL min^−1^. The concentration of the drug encapsulated inside the liposomes was calculated based on a JS‐K or TMZ standard concentration curve.

### Cell Culture

Mouse glioma cancer cells (GL261) were purchased from the Cell Bank of Shanghai Institute of Cell Biology, Chinese Academy of Sciences (Shanghai, China). The mouse brain capillary endothelial cell line bEnd.3 was purchased from Jennio Biotech Co., Ltd. (Guangzhou, China). The mouse hippocampal neurons cell line HT22 was purchased from Procell Life Science & Technology Co.,Ltd. (Wuhan, China). All these cells were cultured in Dulbecco's Modified Eagle's Medium (DMEM) with 10% fetal bovine serum (FBS, Gibco) and 1% penicillin/streptomycin antibiotic (Thermo Fisher Scientific). And GL261 cells were maintained in a 5% CO_2_ cell‐culture incubator at 37 °C with humidified atmosphere.

### Confocal Imaging of Cellular S1P/JS‐K/TMZ/Lipo Uptake and Organelle Distribution

GL261 cells (5000 cells per well) were seeded onto a 96‐well plate in a 37 °C incubator. After overnight incubation, the culture medium was replaced with S1P/JS‐K/TMZ/Lipo in DMEM medium (10 µm). After incubation for different time periods, the treated cells were washed with PBS for three times and then imaged using confocal laser scanning microscopy (CLSM, Ti C2+, Nikon, Japan).

To show the intracellular distribution of S1P/JS‐K/TMZ/Lipo at different time points, S1P/JS‐K/TMZ/Lipo‐treated GL261 cells were incubated with S1P/JS‐K/TMZ/Lipo for 0, 2, 4, 8, 12, or 24 h, respectively, and stained with LysoTracker Red (0.5 µM, Ex: 552 nm). Then the cells were imaged under a confocal microscope. Co‐localization coefficients of the cells were analyzed by an Image‐Pro Plus software (Rockville, MD, USA).

### In Vitro NO Generation and Characterization

In order to detect the generation process of NO in cells incubated with different treatments, the NO fluorescent probe DAF‐FM DA was pre‐treated with the GL261 cells at a final concentration of 5 × 10^−3^ µm. After 20 min incubation in a 37 °C incubator in the dark, the unloaded fluorescent probe was removed by PBS washing for three times. Then, GL261 cells were treated with PBS, JS‐K/Lipo, TMZ/Lipo, S1P/JS‐K/Lipo, or S1P/JS‐K/TMZ/Lipo. An optical microscope (TS100/TS100‐F, Nikon Co., Ltd., Japan) was used to monitor real‐time NO generation at different time periods.

### Cell Viability Measurement and Western Blot Assay

First, to evaluate the cytotoxicity of Lipo, S1P/Lipo, JS‐K/Lipo, TMZ/Lipo, S1P/JS‐K/Lipo, and S1P/JS‐K/TMZ/Lipo, GL261 cells seeded in 96‐well plates at a density of 5 × 10^3^ per well were treated with different concentrations of Lipo, S1P/Lipo, JS‐K/Lipo, TMZ/Lipo, S1P/JS‐K/Lipo, and S1P/JS‐K/TMZ/Lipo (1, 2, 5, 10, 20, and 40 µm), respectively. Then, the cell viabilities were determined using CCK‐8 assay. Specifically, after incubation for 0, 2, 4, 6, 8, 10, 12, and 24 h, CCK‐8 solution (10 µL, 5 mg mL^−1^) was added to each well with incubation for another 1.5 h at 37 °C. Then the corresponding absorbance at 570 nm was measured by a microplate reader (Thermo‐Scientific, Multiskan FC, USA). The cell viability was calculated in comparison with untreated cells.

Cell lysates were collected in cell lysis buffer for Western and immunoprecipitation (20 mm Tris, pH 7.5, 150 mm NaCl, 1% Triton X‐100, sodium pyrophosphate, *β*‐glycerophosphate, EDTA, Na_3_VO_4,_ leupeptin) and then centrifuged at 14 000 rpm and 4 °C to obtain total protein. Equal amounts of proteins were boiled and fractionated by 4%–20% sodium dodecyl sulfate‐polyacrylamide gel electrophoresis (SDS‐PAGE) gel. Primary antibodies against the following proteins were used: LC3, p62, and PTEN. GAPDH was used as a reference protein. Signal was detected using Western ECL Substrate (Tanon, China).

### Mitochondrial Membrane Potential Measurements

To quantify the MMP changes of GL261 cells after different treatments, mitochondrial membrane potential probe JC‐1 was used. Specifically, GL261 cells were cultured in 6‐well culture plate at the density of 2 × 10^5^ per well at 37 °C overnight. Then, the cells were treated with PBS, JS‐K/Lipo, TMZ/Lipo, S1P/JS‐K/Lipo, or S1P/JS‐K/TMZ/Lipo for 24 h. Subsequently, the treated cells were stained by JC‐1 MMP assay kit and quantitatively measured by flow cytometry.

### Imaging and Evaluation of Cellular ROS Levels

Intracellular ROS levels were measured using the ROS assay kit. Specifically, GL261 cells were cultured in confocal dishes (2 × 10^5^ cells) and divided into five groups: Control, JS‐K/Lipo, TMZ/Lipo, S1P/JS‐K/Lipo, and S1P/JS‐K/TMZ/Lipo. GL261 cells in Control group were incubated with PBS, and others were treated with 20 µm JS‐K/Lipo, TMZ/Lipo, S1P/JS‐K/Lipo, and S1P/JS‐K/TMZ/Lipo, respectively. After 24 h incubation, the cells were washed with PBS for three times, and then incubated with fresh culture medium. Finally, the cells were loaded with DCFH‐DA (10 µm) for 20 min in the dark (at 37 °C in 5% CO_2_). The cells were observed under LSCM.

### Detection of Immunogenic Cell Death of GL261 Cells

GL261 cells were cultured in 24‐well culture plate at the density of 2 × 10^5^ per well. After 24 h incubation, GL261 cells were treated with PBS, JS‐K/Lipo, TMZ/Lipo, S1P/JS‐K/Lipo, or S1P/JS‐K/TMZ/Lipo for 24 h. Afterward, calreticulin (CRT) expressed on the GL261 cell membrane surface was localized by immunofluorescence staining, subsequently imaged and analyzed by LSCM. Besides, cell culture medium of GL261 cells after different treatments was collected and the concentrations of HMGB1 and ATP in the collected medium were qualified by ELISA kits (Sangon, China).

### RNA Sequencing

GL261 cells (5 × 10^6^) with different treatments were treated with TRIzol Reagent (Invitrogen, USA) at 8 h after co‐incubation. The experiments were independently performed three times for each group. RNA sequencing was performed by NovaSeq 6000 sequencer. After the disassembly of RNA‐seq data, the raw reads were filtered by SOAPnuke. The HISAT2 software was used to map the sequencing data to the human reference genome (GRCh38) using default parameters. The feature Counts software was used to quantify gene expression at the gene level. A heat map of gene expression levels in the different samples was drawn with heatmap (v1.0.8). Briefly, differential expression analysis was performed using edgeR with a *q* value ≤ 0.05. To gain insight into the change in phenotype, GO and KEGG enrichment analysis of differentially expressed genes was performed with clusterProfiler based on the Benjamini‐Hochberg test. The significance levels of terms and pathways were corrected with Bonferroni's correction based on a rigorous *q* value threshold (*q* value ≤ 0.05).

### Animal Model

For animal experiments, female BALB/c mice (20 ± 2 g) were purchased from Shanghai Laboratory Animals Center (Shanghai, China). All mice were 6–8 weeks old and kept under specific pathogen free (SPF) conditions in a 12 h light‐dark cycle, a room temperature of 20—22 °C, and a humidity of 40%–60% for at least 1 week to adapt the experimental conditions. All animal experiments, animal care and animal model protocols were approved by the Animal Care Committee of Southeast University (NO. 20220216009) in accordance with the Regulations for the Administration of Affairs Concerning Experimental Animals of China. To establish in situ glioma tumor‐bearing mouse model, the mouse glioma cancer cell GL261 was used. In brief, 1 × 10^6^ GL261 cells were stereotactic intracranial implanted into the brain parenchyma of mouse through a craniotomy open window to establish tumors. At 7 days post implantation, mice with glioma in brain regions were confirmed by magnetic resonance imaging system (BRUKER, USA). The mice with similar area of glioma lesion were then randomly divided into different groups according to the experiment requirement.

### Biodistribution of S1P/JS‐K/TMZ/Lipo in a Tumor‐Bearing Mouse Model

To characterize brain tumor targeting capability of S1P/JS‐K/TMZ/Lipo, mice were randomly divided into five groups (*n* = 8 per group): Group 1, mouse model injected with DiR‐S1P/JS‐K/TMZ/Lipo (100 µL); Group 2, mice injected with DiR‐TMZ/Lipo (100 µL); Group 3, mice injected with DiR‐S1P/Lipo (100 µL); Group 4, mice injected with DiR‐JS‐K/Lipo (100 µL); and Group 5, mice injected with PBS (100 µL). At pre‐determined time interval post administration via tail vein, the mice were imaged using an in vivo optical imaging system (IVIS Spectrum Imaging System, Caliper Life Sciences, USA). To study tissue biodistribution, mice in each group were sacrificed post injection and the major organs (heart, liver, spleen, lung, kidney) excised and captured by a NIR fluorescence in vivo imaging system.

### In Vivo Antitumor Efficacy of S1P/JS‐K/TMZ/Lipo

To evaluate the therapeutic effect of S1P/JS‐K/TMZ/Lipo, the mice were randomly divided into four groups as follows: PBS, JS‐K/Lipo, TMZ/Lipo and S1P/JS‐K/TMZ/Lipo. All the mice were intravenously injected with PBS (100 µL), JS‐K/Lipo (100 µL), TMZ/Lipo (100 µL) and S1P/JS‐K/TMZ/Lipo (100 µL). Tail vein injections treatments were performed every 2 days, for a total of five injections. Body weight was recorded every other day for 16 days. Finally, mice were sacrificed to collect tumors with major organs (heart, liver, spleen, lung, and kidney) to analyze T cell infiltration and tumor apoptosis with different treatments. First of all, the tissue sections of 3 µm thickness from tumors were soaked in acetone for 15 min and then gently washed with PBS. Then, the sections were blocked with 5% BSA and incubated with antibodies against FITC labeled anti‐mouse CD11c (1:250, Biolegend), and FITC labeled anti‐mouse Foxp3 (1:250, Biolegend), FITC labeled anti‐mouse CD4 (1:250, Biolegend), FITC labeled anti‐mouse CD8 (1:250, Biolegend) for 4 h. Next, sections were gently washed three times by PBS and stained with DAPI. Finally, CLSM was applied to collect images.

### Statistical Analysis

Quantitative data were presented as means ± standard deviation (SD) from sample numbers (*n*). Data from experiments were analyzed using GraphPad Prism 9. Statistical comparisons were made by unpaired Student's *t*‐test (between two groups) and one‐way ANOVA (for multiple comparisons). ^*^ *p* value < 0.05 was considered statistically significant, ^**^ *p* < 0.01 and ^***^ *p* < 0.001 were extremely significant, NS was considered no significant difference. For quantitative analysis in fluorescence intensity for confocal images, Image J software (National Institutes of Health, USA) was used for densitometric analysis.

## Conflict of Interest

The authors declare no conflict of interest.

## Author Contributions

F.Y. and Y.L. conceived the idea. F.Y. and N.G. supervised this project. Y.L. and L.C. performed all the experiments and analyzed data. Y.L. and W.L.M. performed the fabricated and characterization of S1P/JS‐K/TMZ/Lipo and other control liposomes. L.C. assisted with the flow cytometry studies. L.C. and X.W. provided help in the orthotropic glioma mice model experiments. X.W. help to perform in the Western Blot analysis of protein. X.W., W.L.M., H.T.X., and T.D.C. help to perform the GL261 cells experiments. Y.L., L.C., T.D.C., and F.Y. wrote the manuscript and revised according to the comments from other coauthors.

## Supporting information

Supporting Information

Supporting Information

## Data Availability

The data that support the findings of this study are available from the corresponding author upon reasonable request.
