# Supplementary material for: In Situ Nitric Oxide Gas Nanogenerator Reprograms Glioma Immunosuppressive Microenvironment
Source: Adv Sci (Weinh). 2023 Apr 21;10(18):2300679. doi: 10.1002/advs.202300679 (PMC10288280; doi:10.1002/advs.202300679)
Supplement: Supplementary file 1 — Supporting Information [file ADVS-10-2300679-s002.pdf]

Supporting Information

In Situ Nitric Oxide Gas Nanogenerator Reprograms Glioma Immunosuppressive Microenvironment

Yang Liu, Lin Cui, Xiao Wang, Weiling Miao, Yongxu Ju, Tiandong Chen, Huiting Xu, Ning Gu, and Fang Yang**

State Key Laboratory of Bioelectronics, Jiangsu Key Laboratory for Biomaterials and Devices
School of Biological Sciences and Medical Engineering
Southeast University
Nanjing 210096, P. R. China

E-mail: yangfang2080@seu.edu.cn (Fang Yang); guning@seu.edu.cn (Ning Gu)

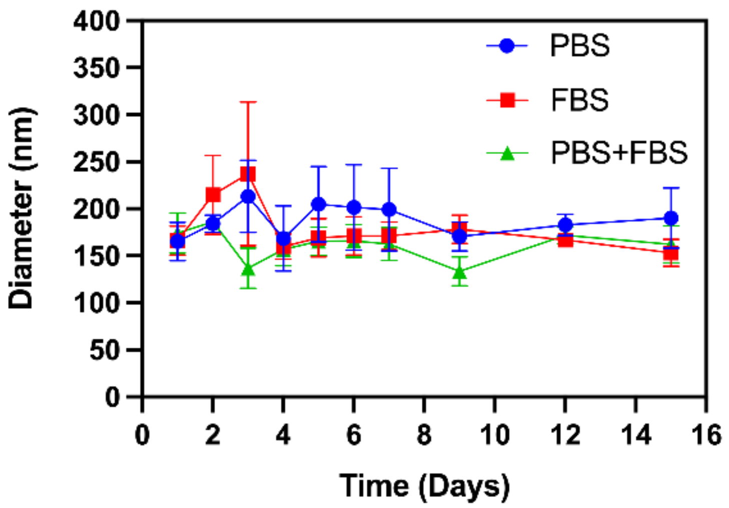

Figure S1. The hydrodynamic diameter of S1P/JS-K/TMZ/Lipo in phosphate-buffered saline (PBS, pH 7.4), 10% fetal bovine serum (FBS) or a combination of PBS (pH 7.4) and 10% FBS over 15 days. Error bar, means ± SD (n=3).

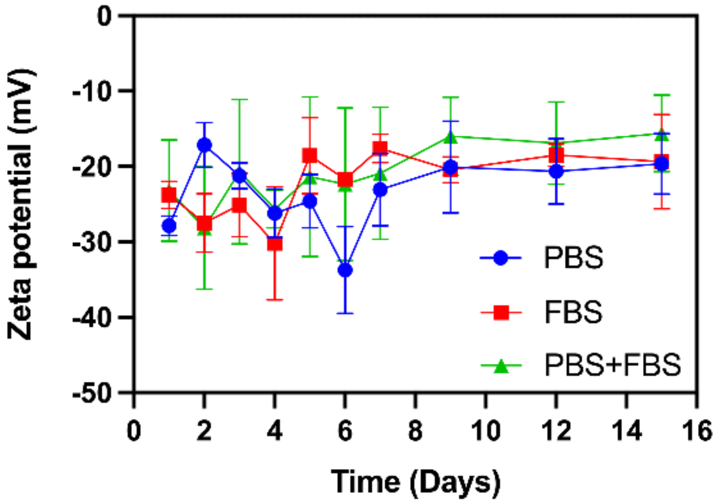

Figure S2. The zeta potential of S1P/JS-K/TMZ/Lipo in phosphate-buffered saline (PBS, pH 7.4), 10% fetal bovine serum (FBS) or a combination of PBS (pH 7.4) and 10% FBS over 15 days. Error bar, means ± SD (n=3).

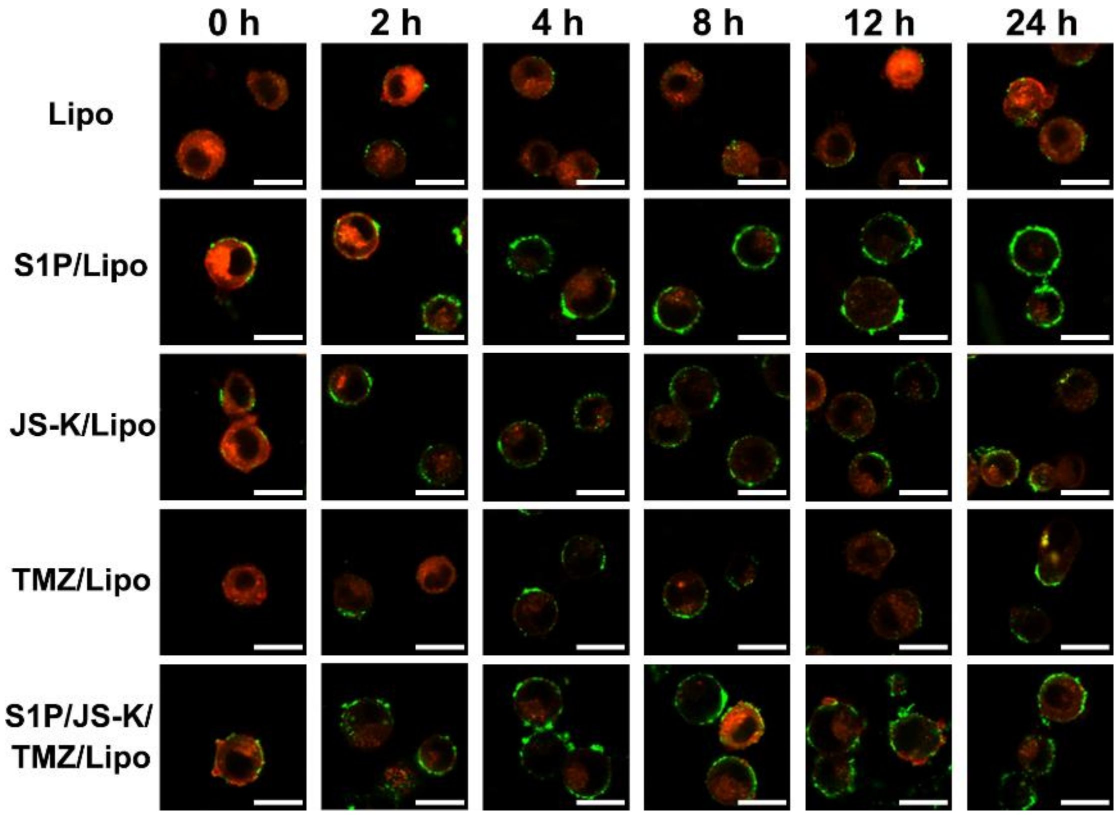

Figure S3. CLSM images of the cellular uptake of Lipo, S1P/Lipo, JS-K/Lipo, TMZ/Lipo or S1P/JS-K/TMZ/Lipo by GL261 cells at different time points (0, 2, 4, 8, 12, and 24 h). GL261 Cells were stained with DiI (orange), and liposome formulations were stained by DiO (green) (Scale bars, 20 μm).

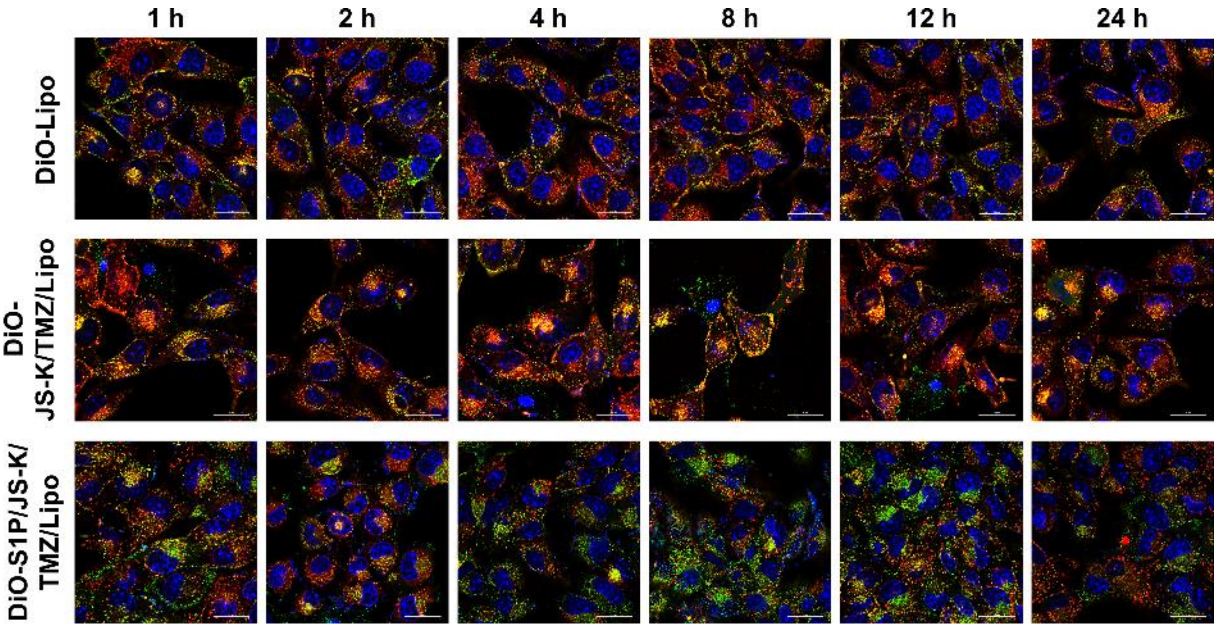

Figure S4. CLSM images of the lysosomal escape of different treatments (Lipo, JS-K/TMZ/Lipo and S1P/JS-K/TMZ/Lipo) co-incubated with GL261 cells for different times. The co-localization of different treatments with lysosomes was observed at different time points (1, 2, 4, 8, 12 and 24 h). GL261 cell lysosomes were stained with LysoTracker (Red) for labeling, and liposome formulations were stained with DiO (Green) (Scale bars, 10 μm). Pearson coefficients were calculated by ImageJ.

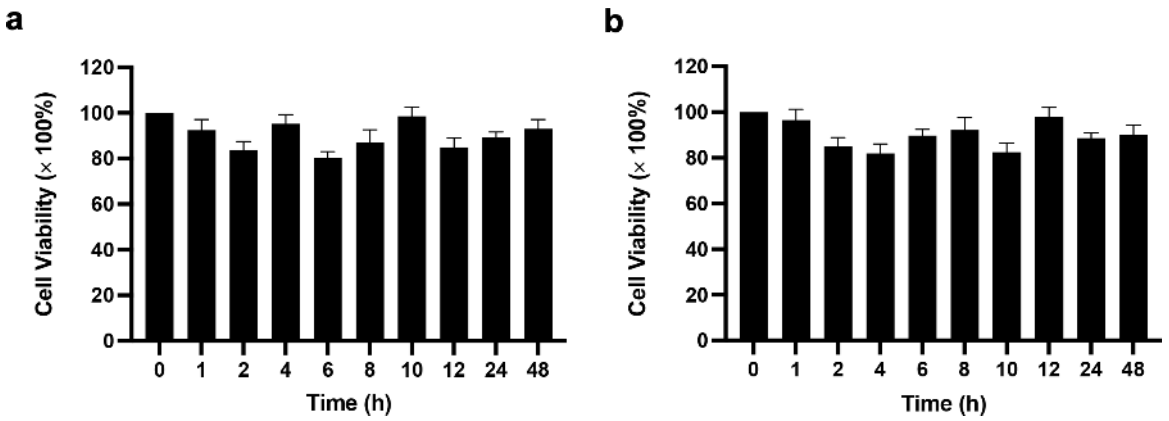

Figure S5. Cell viability of a) bEnd.3 and b) HT22 cells incubated with S1P/JS-K/TMZ/Lipo (TMZ: 500 μg/ml, JS-K: 180 μg/ml) for different time periods.

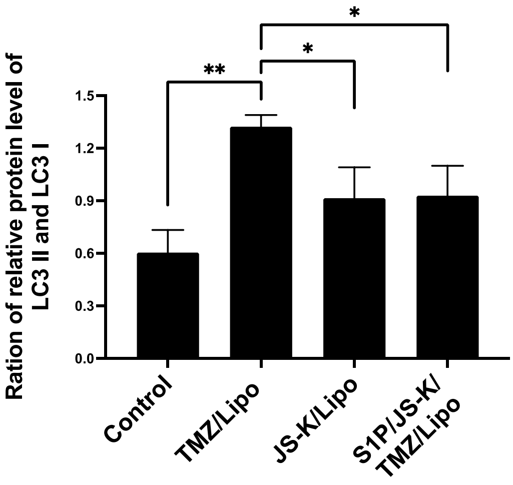

Figure S6. Statistical analysis of the LC3 II/LC3 I ratio among the four groups. Data represent mean ± SD, and three independent experiments (n = 3) were performed. The statistical significance is indicated by *p < 0.05 and **p < 0.01 determined using one-way analysis of variance (ANOVA) followed by Student's t-test (two-tailed).

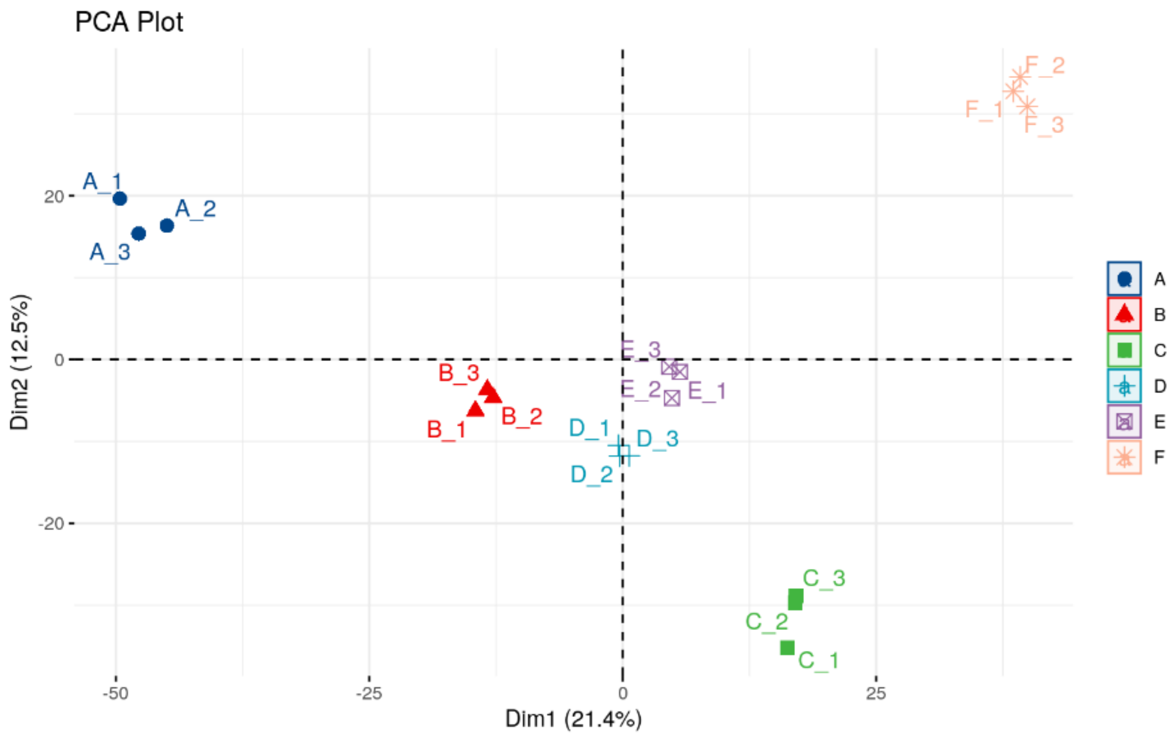

Figure S7. PCA of transcriptomes of DEGs in GL261 cells with different treatments, including (A) Control group, (B) Lipo group, (C) S1P/Lipo, (D) JS-K/Lipo, (E) TMZ/Lipo and (F) S1P/JS-K/TMZ/Lipo.

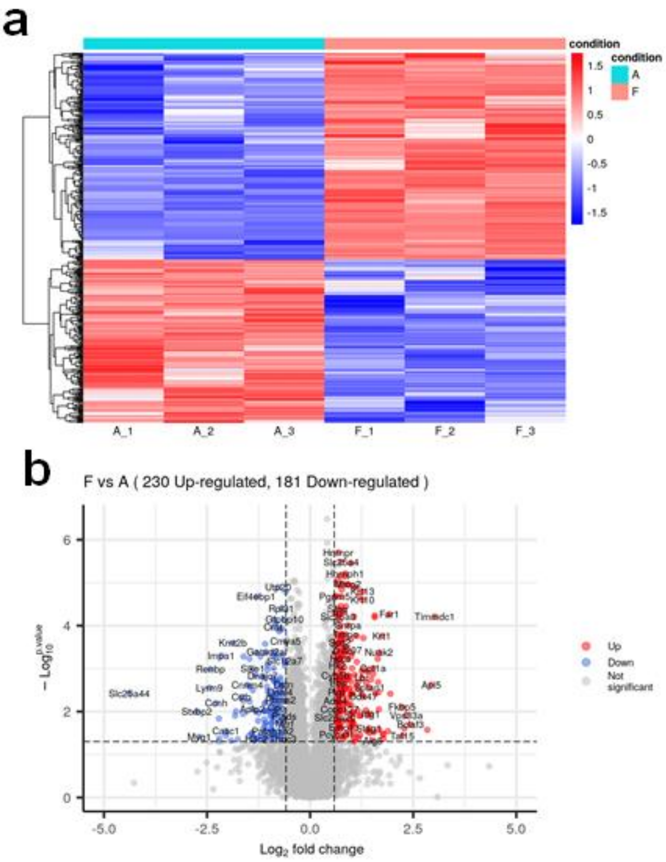

Figure S8. (**a)** Heatmap and (**b**) Volcano plot of DEGs in the S1P/JS-K/TMZ/Lipo *vs.* Control group.

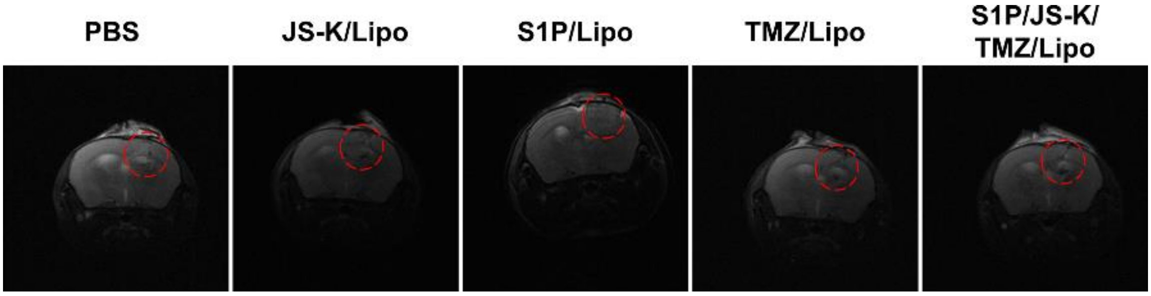

Figure S9. Representative magnetic resonance imaging (MRI) images of mice assigned to different treatments (PBS, JS-K/Lipo, S1P/Lipo, TMZ/Lipo and S1P/JS-K/TMZ/Lipo) 7 days after orthotopic injection of tumor cells. Note that the tissue in the red dotted circle in the figure were in situ gliomas.

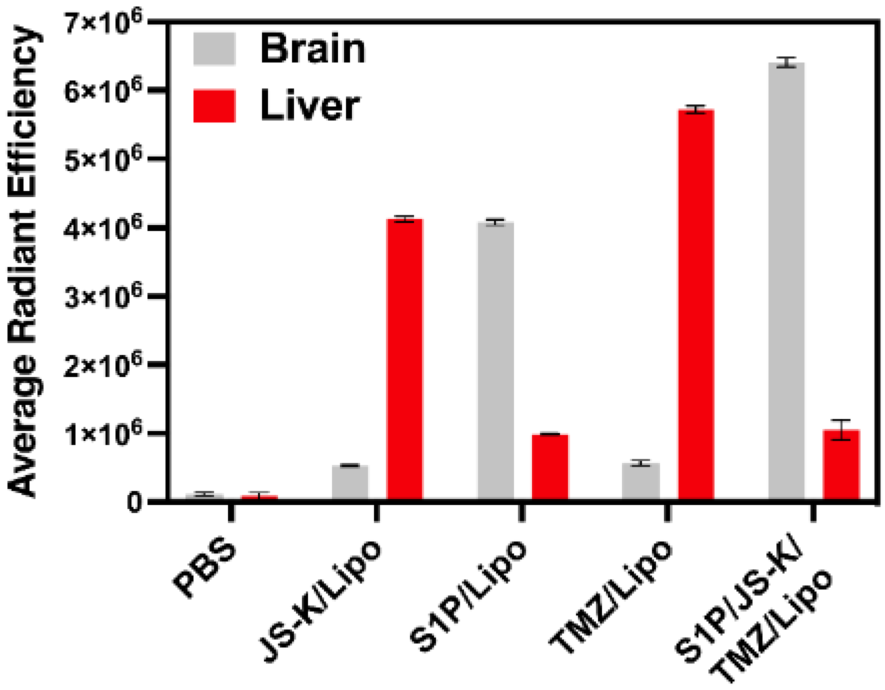

Figure S10. The fluorescence intensity in the brain and liver of mice after tail vein injection of different treatments (PBS, DiR-JS-K/Lipo, DiR-S1P/Lipo, DiR-TMZ/Lipo and DiR-S1P/JS-K/TMZ/Lipo).

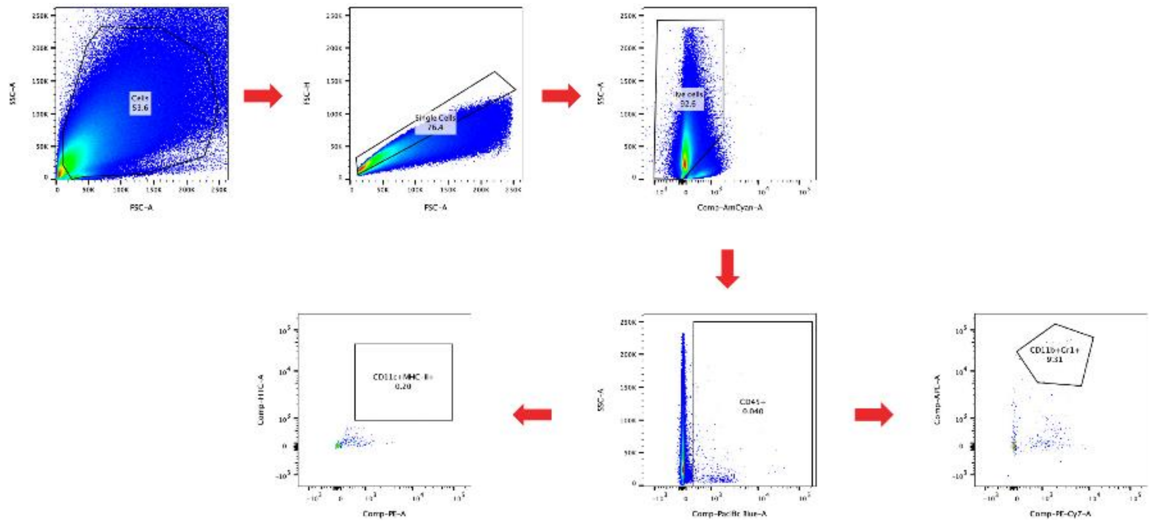

Figure S11. Gating strategy for flow cytometric analysis of extracellular makers on DC cells in tumors.

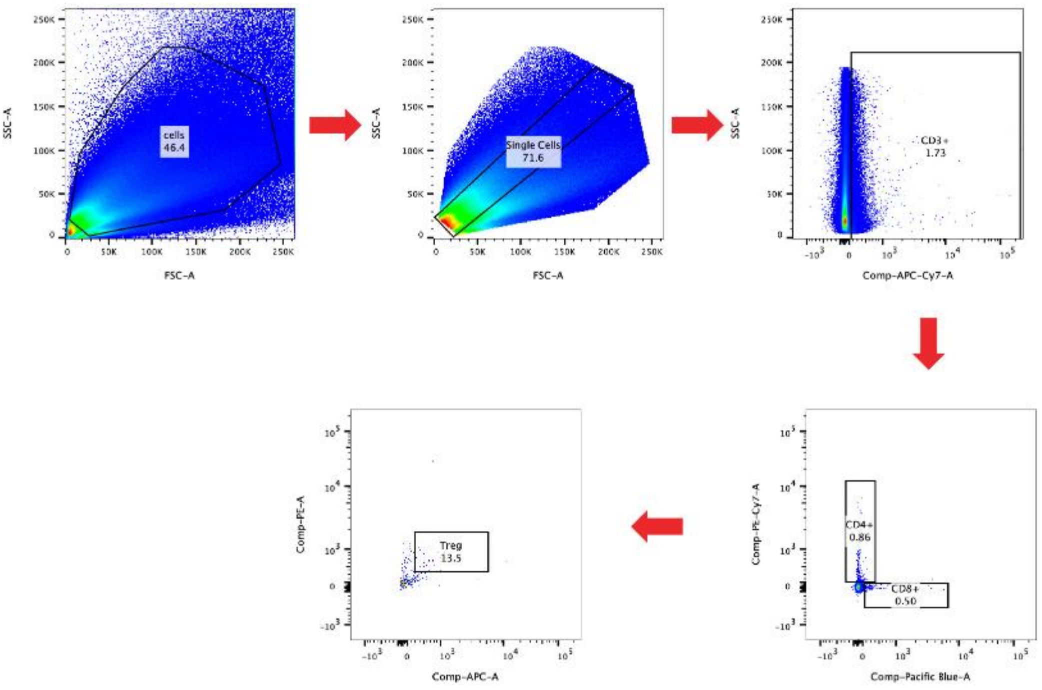

Figure S12. Gating strategy for flow cytometric analysis of extracellular makers on T cells in tumors.

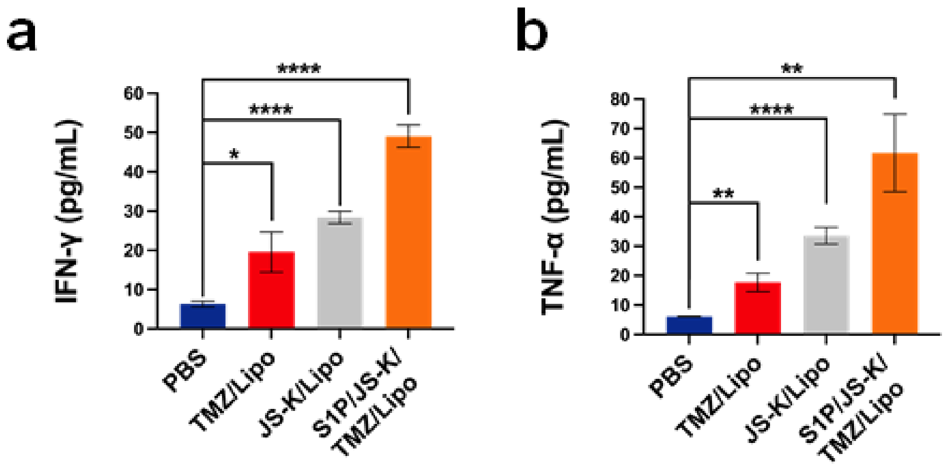

Figure S13. The levels of immune-related cytokines, IFN-γ (**a**) and TNF-α (**b**) in the serum of tumor-bearing mice were first measured by ELISA kits on day 12 after tail vein injection with different treatments.

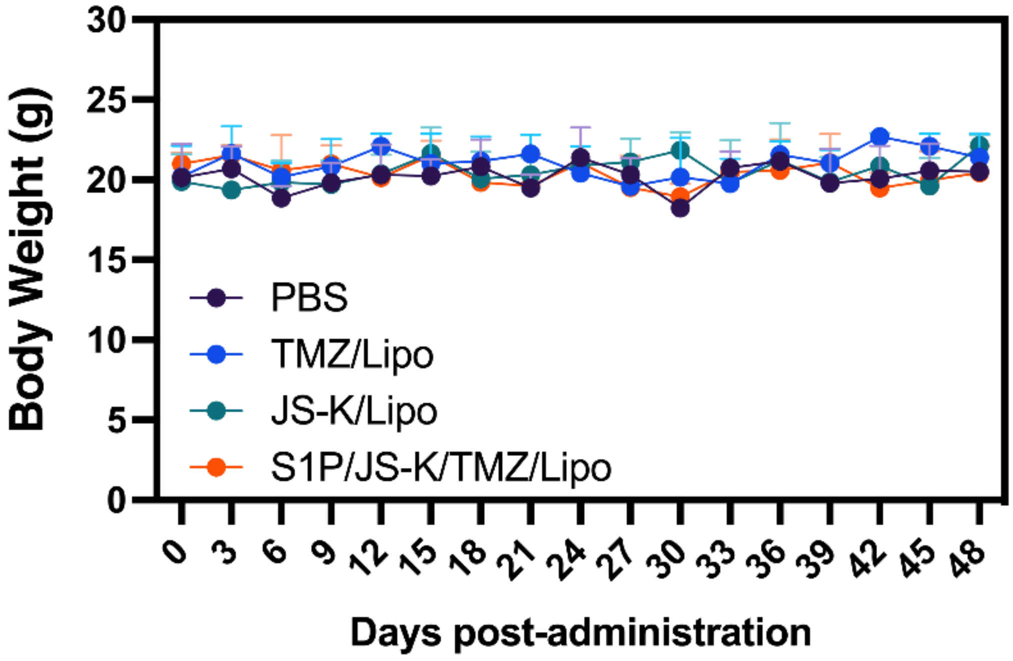

Figure S14. Change in body weight of mice treated with the different formulations.

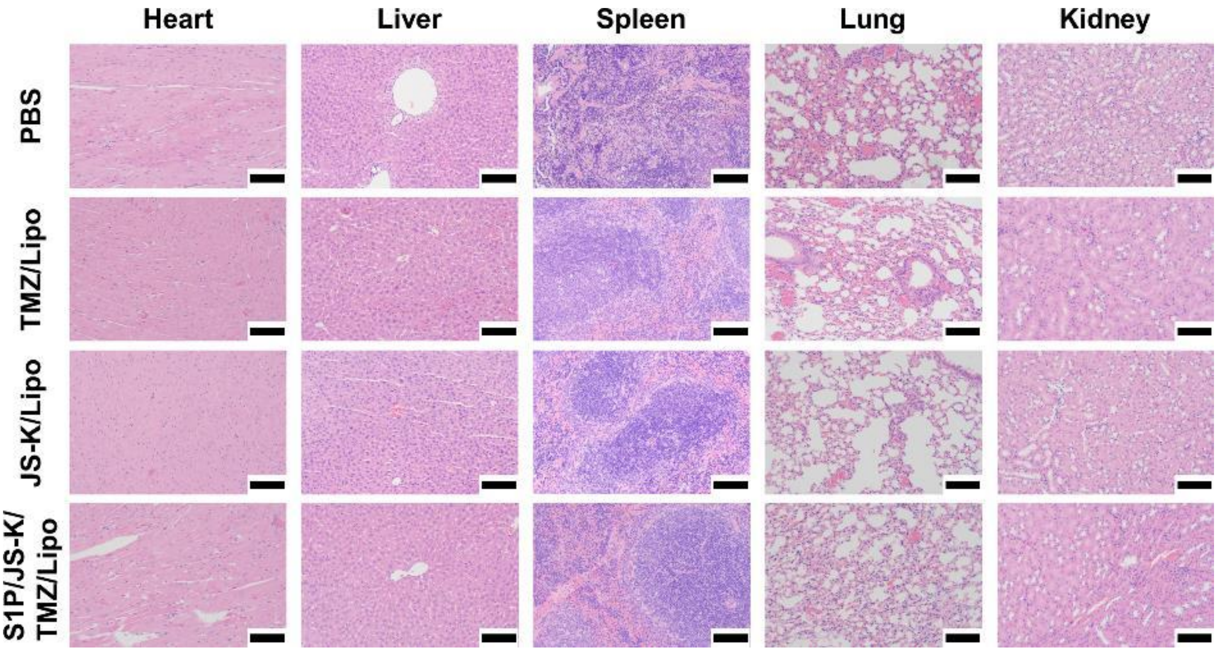

Figure S15. H&E staining images for evaluating biological safety of different liposome formulations (Scale bars, 100 μm).
