# Supplementary figures and images for: In Situ Nitric Oxide Gas Nanogenerator Reprograms Glioma Immunosuppressive Microenvironment
Source: Adv Sci (Weinh). 2023 Apr 21;10(18):2300679. doi: 10.1002/advs.202300679 (PMC10288280; doi:10.1002/advs.202300679)

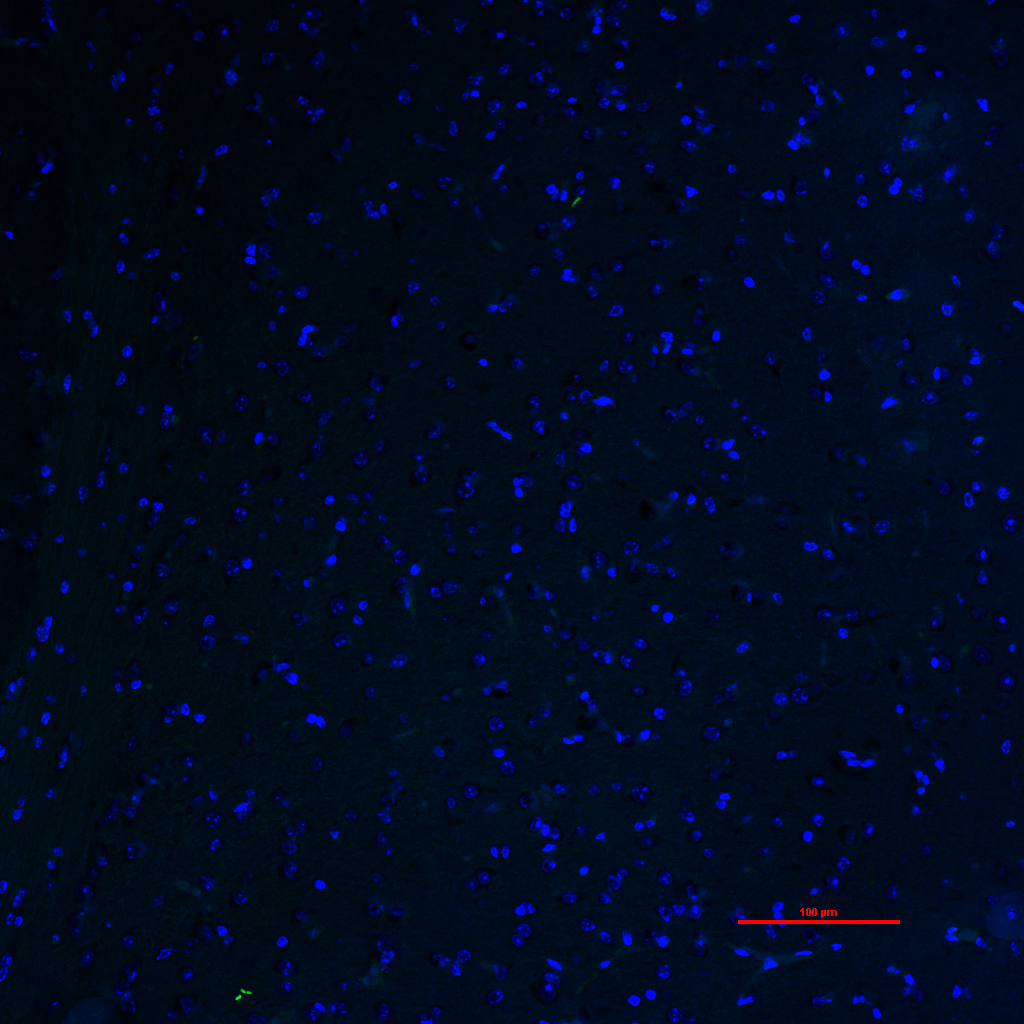

Supplement: Supplementary file 2 — Supporting Information [file ADVS-10-2300679-s001.zip › JS-K_Lipo_RGB.tif]

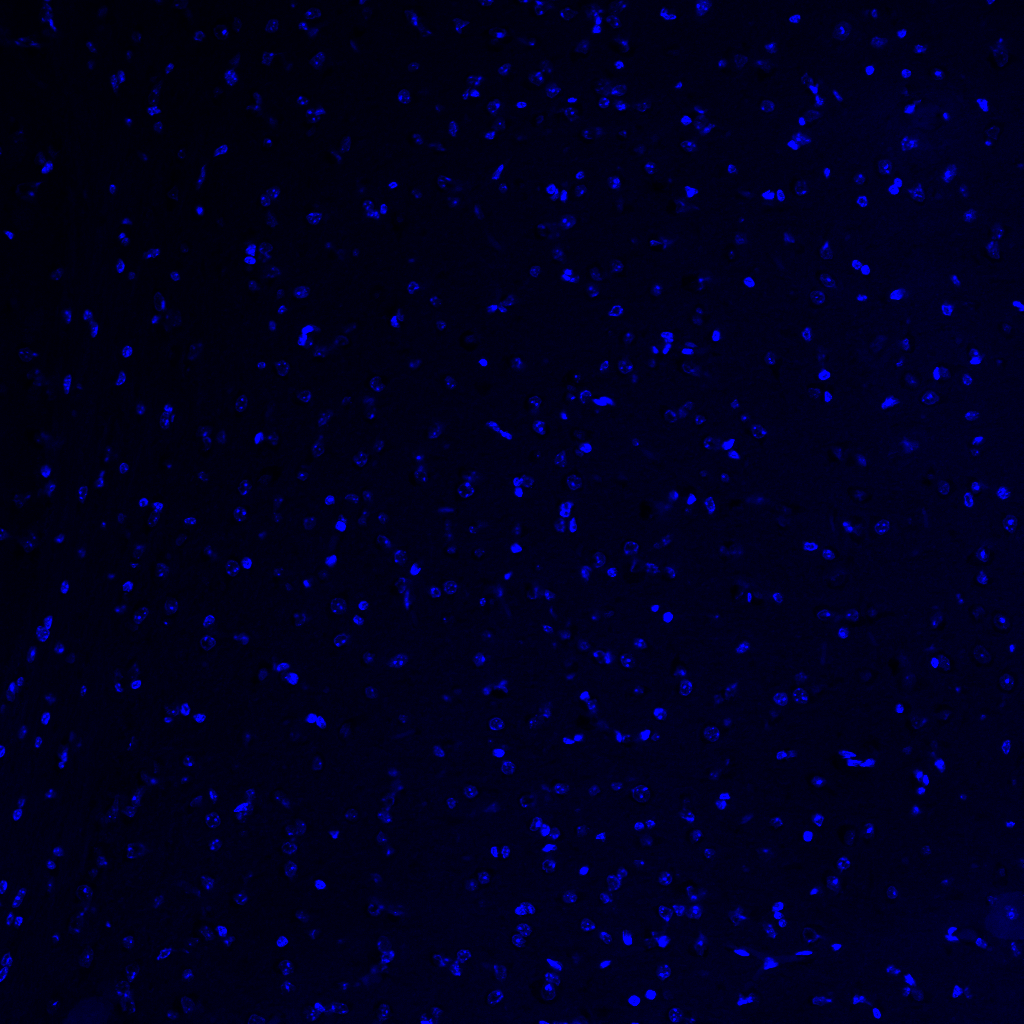

Supplement: Supplementary file 2 — Supporting Information [file ADVS-10-2300679-s001.zip › JS-K_Lipo_RGB_DAPI.tif]

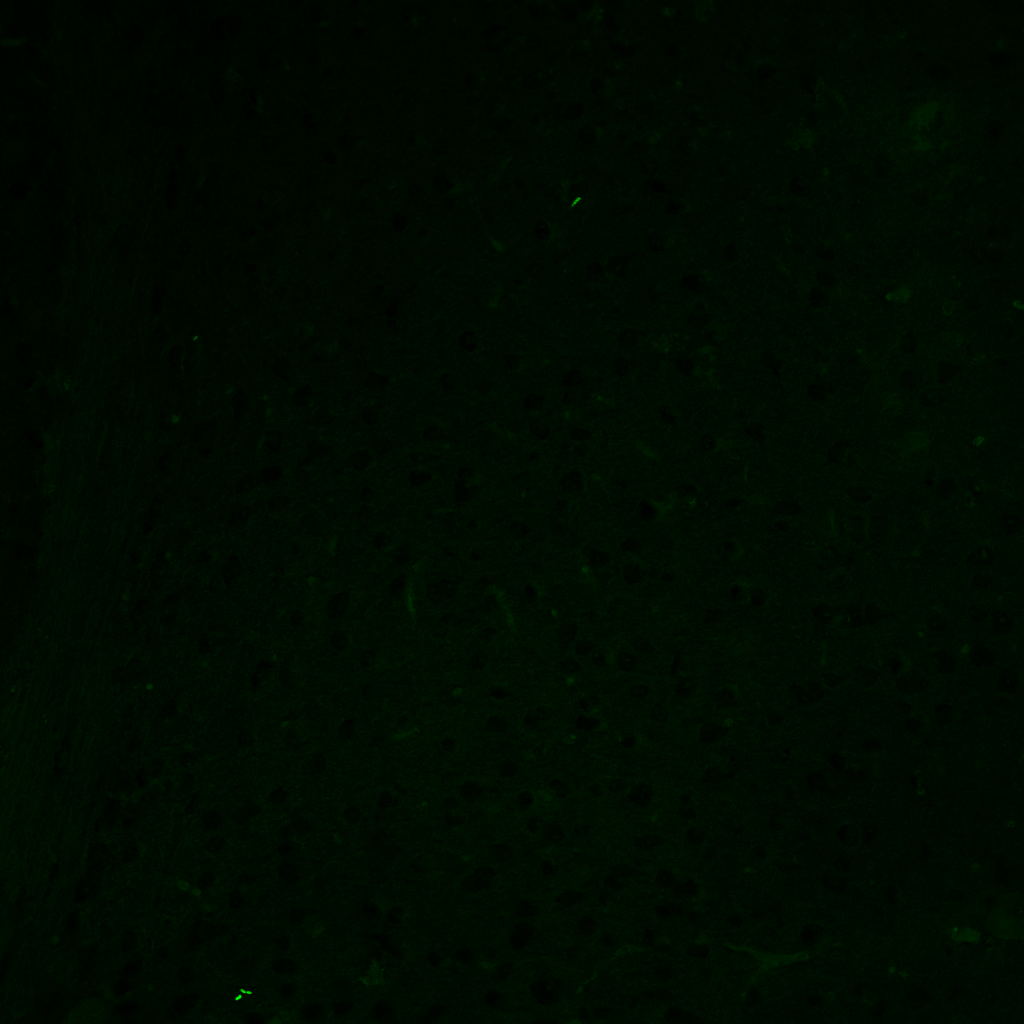

Supplement: Supplementary file 2 — Supporting Information [file ADVS-10-2300679-s001.zip › JS-K_Lipo_RGB_FITC.tif]

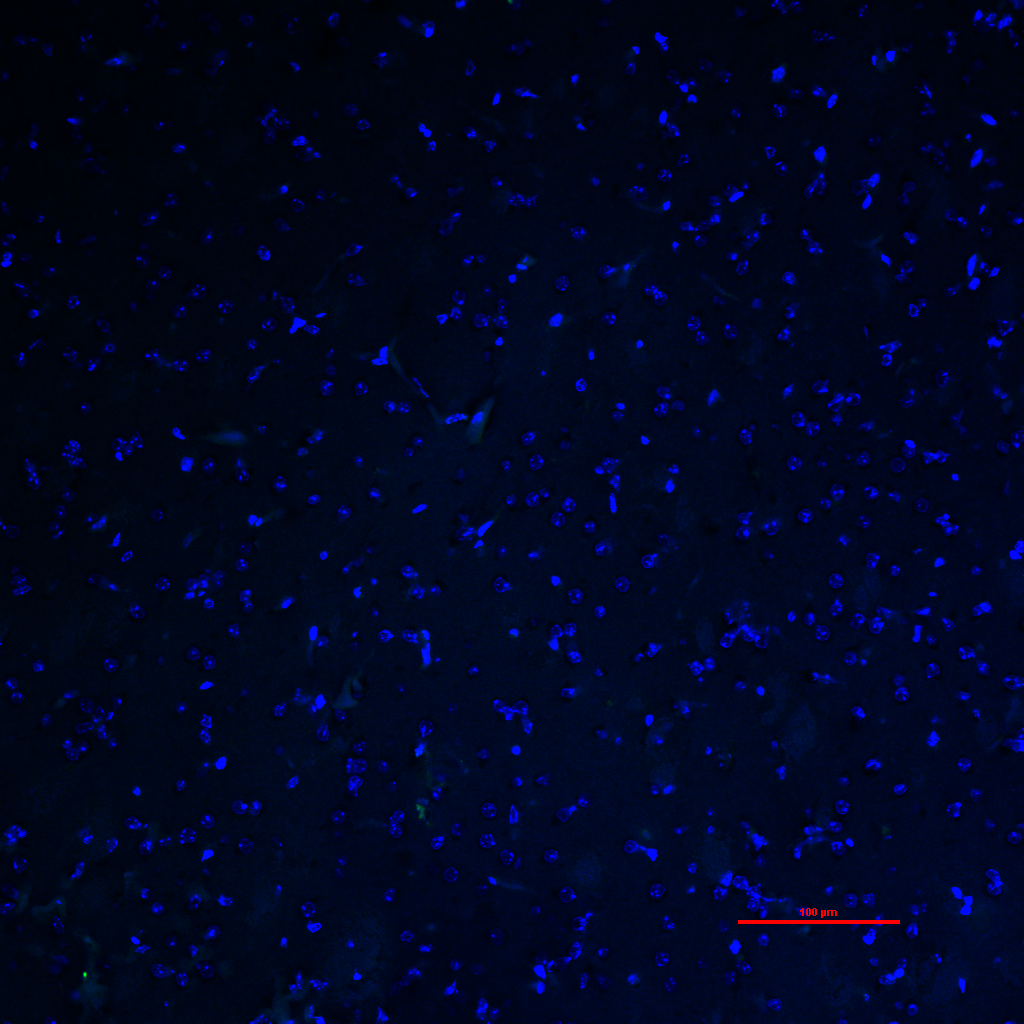

Supplement: Supplementary file 2 — Supporting Information [file ADVS-10-2300679-s001.zip › PBS_RGB.tif]

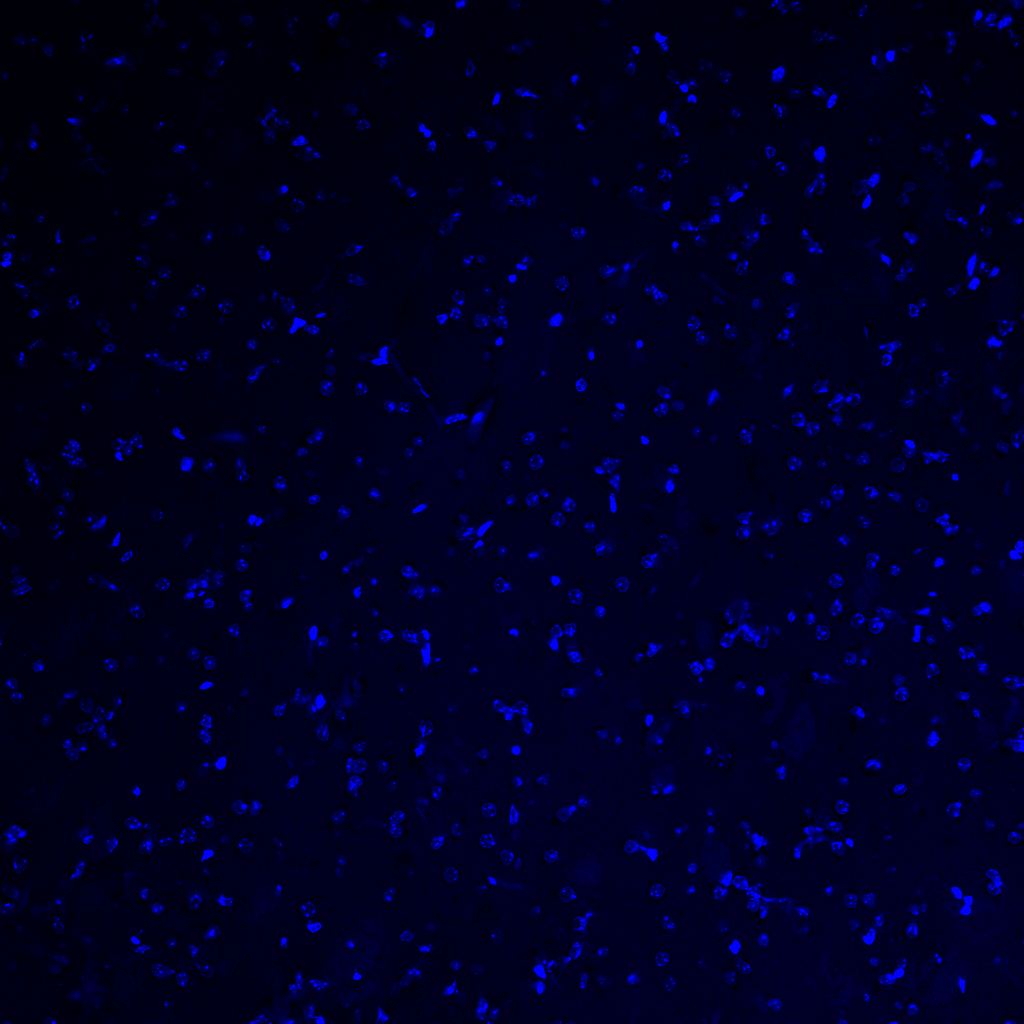

Supplement: Supplementary file 2 — Supporting Information [file ADVS-10-2300679-s001.zip › PBS_RGB_DAPI.tif]

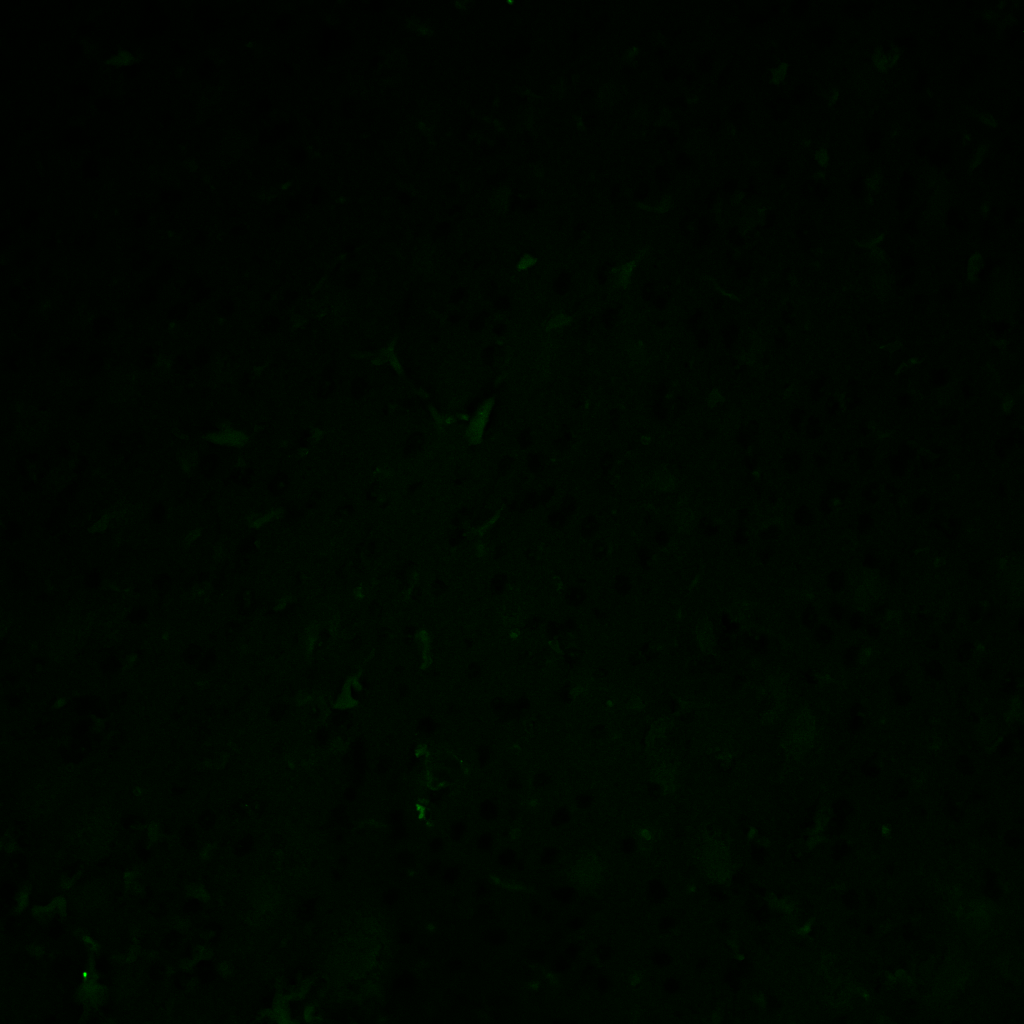

Supplement: Supplementary file 2 — Supporting Information [file ADVS-10-2300679-s001.zip › PBS_RGB_FITC.tif]

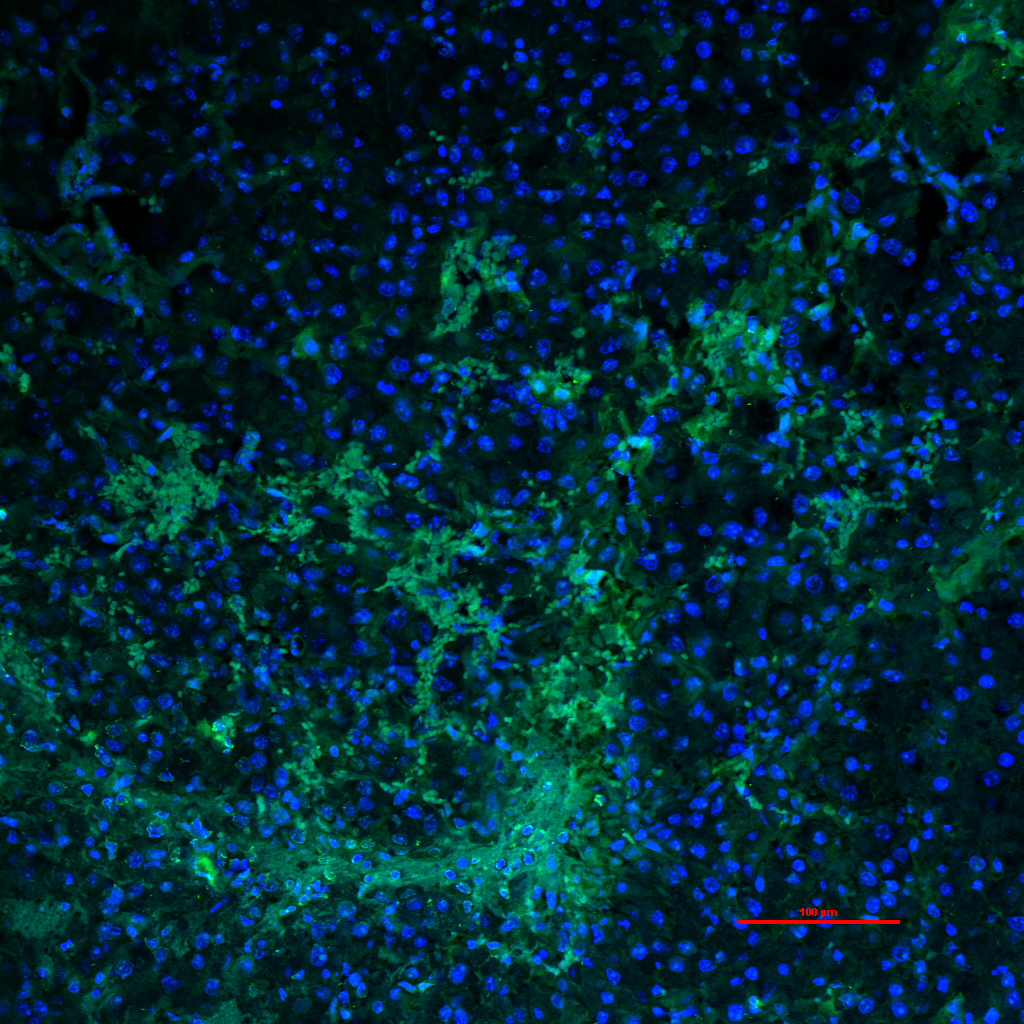

Supplement: Supplementary file 2 — Supporting Information [file ADVS-10-2300679-s001.zip › S1P_JS-K_TMZ_Lipo_RGB.tif]

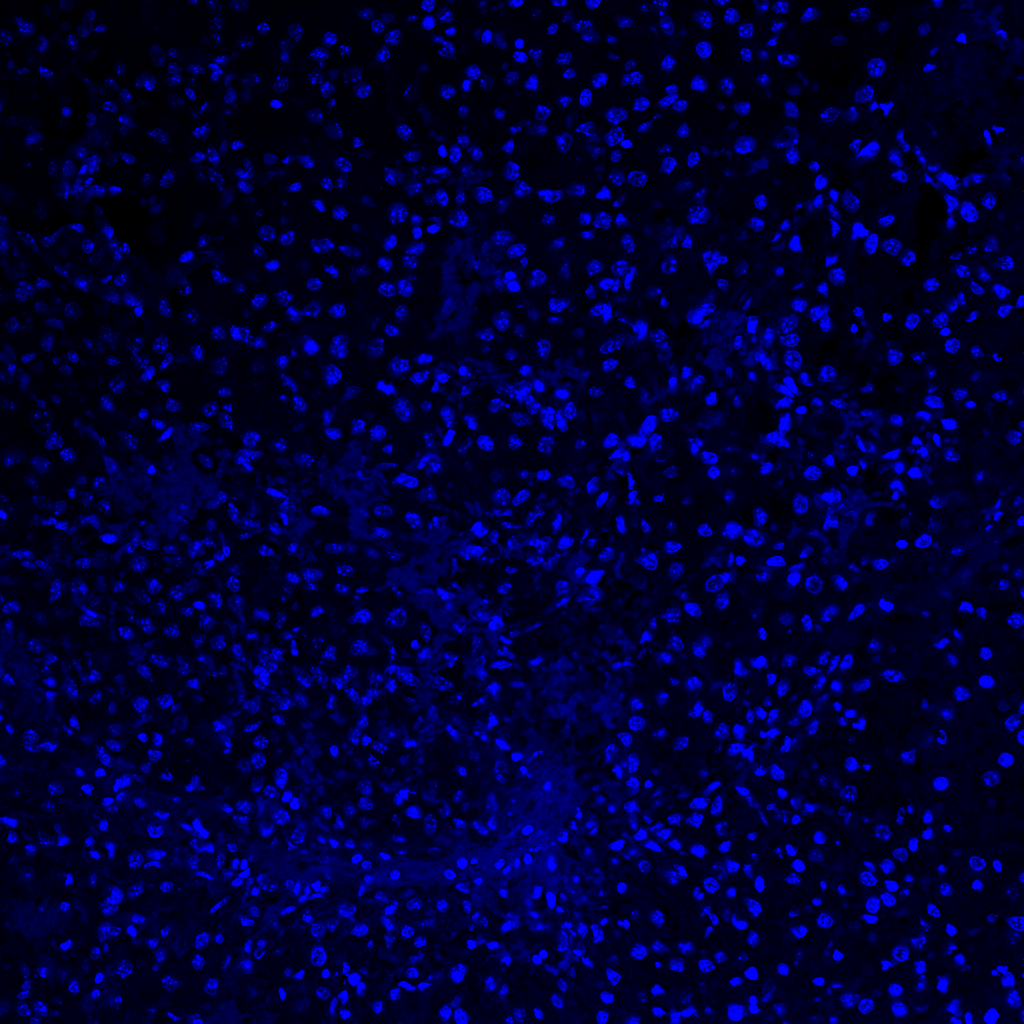

Supplement: Supplementary file 2 — Supporting Information [file ADVS-10-2300679-s001.zip › S1P_JS-K_TMZ_Lipo_RGB_DAPI.tif]

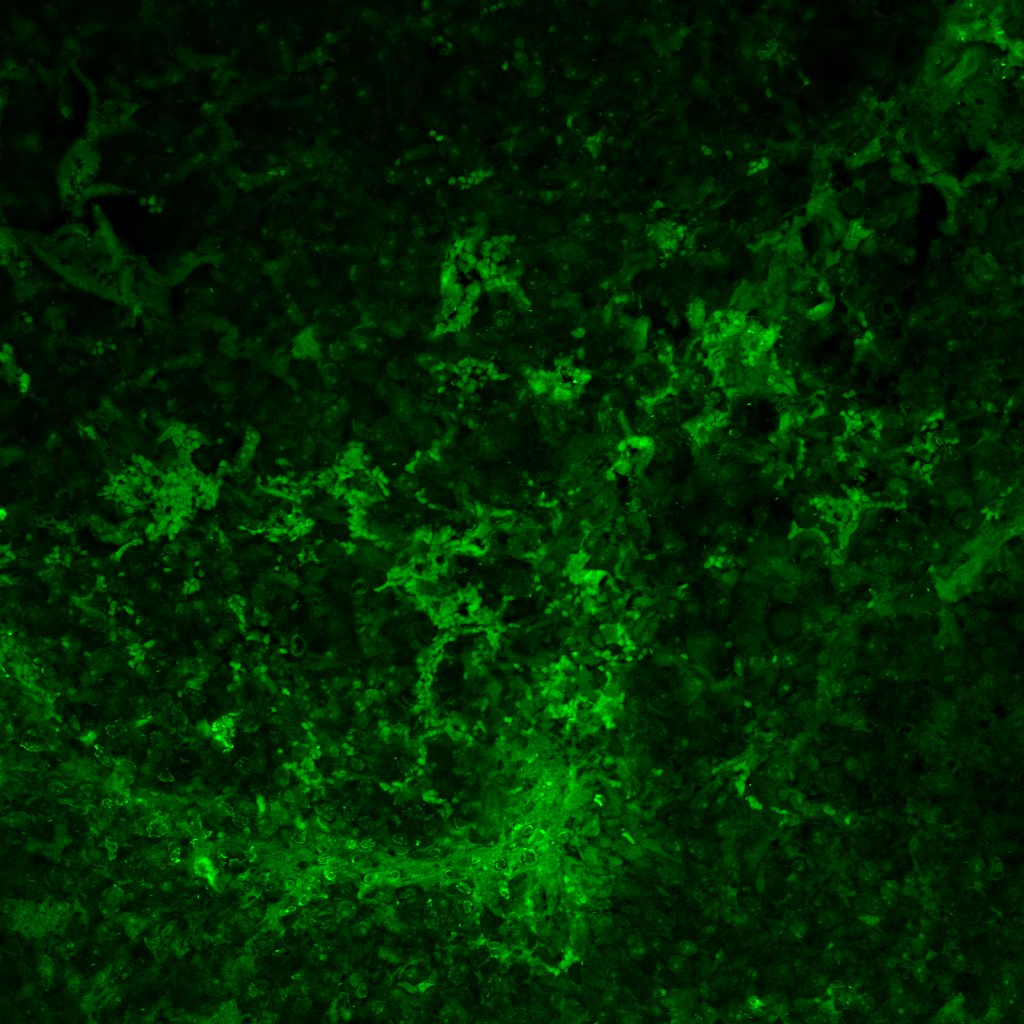

Supplement: Supplementary file 2 — Supporting Information [file ADVS-10-2300679-s001.zip › S1P_JS-K_TMZ_Lipo_RGB_FITC.tif]

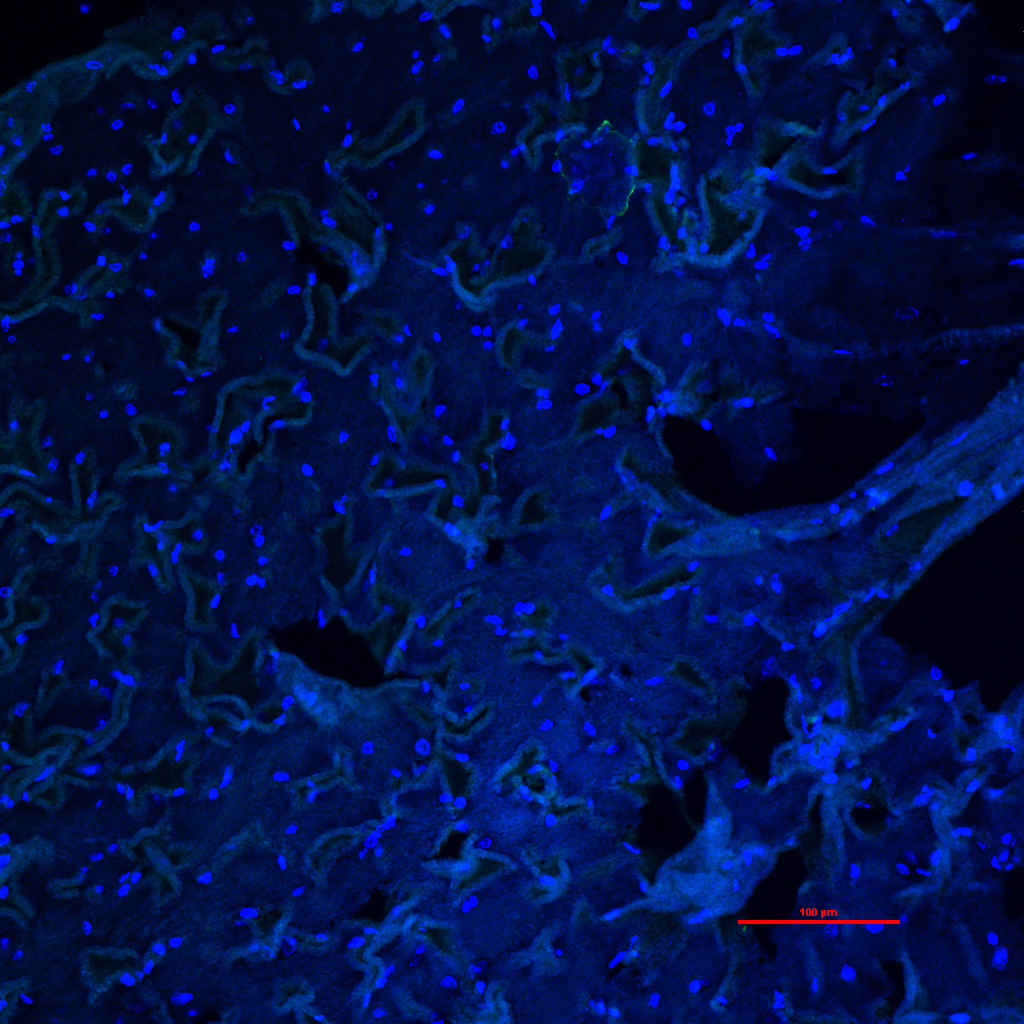

Supplement: Supplementary file 2 — Supporting Information [file ADVS-10-2300679-s001.zip › TMZ_Lipo_RGB.tif]

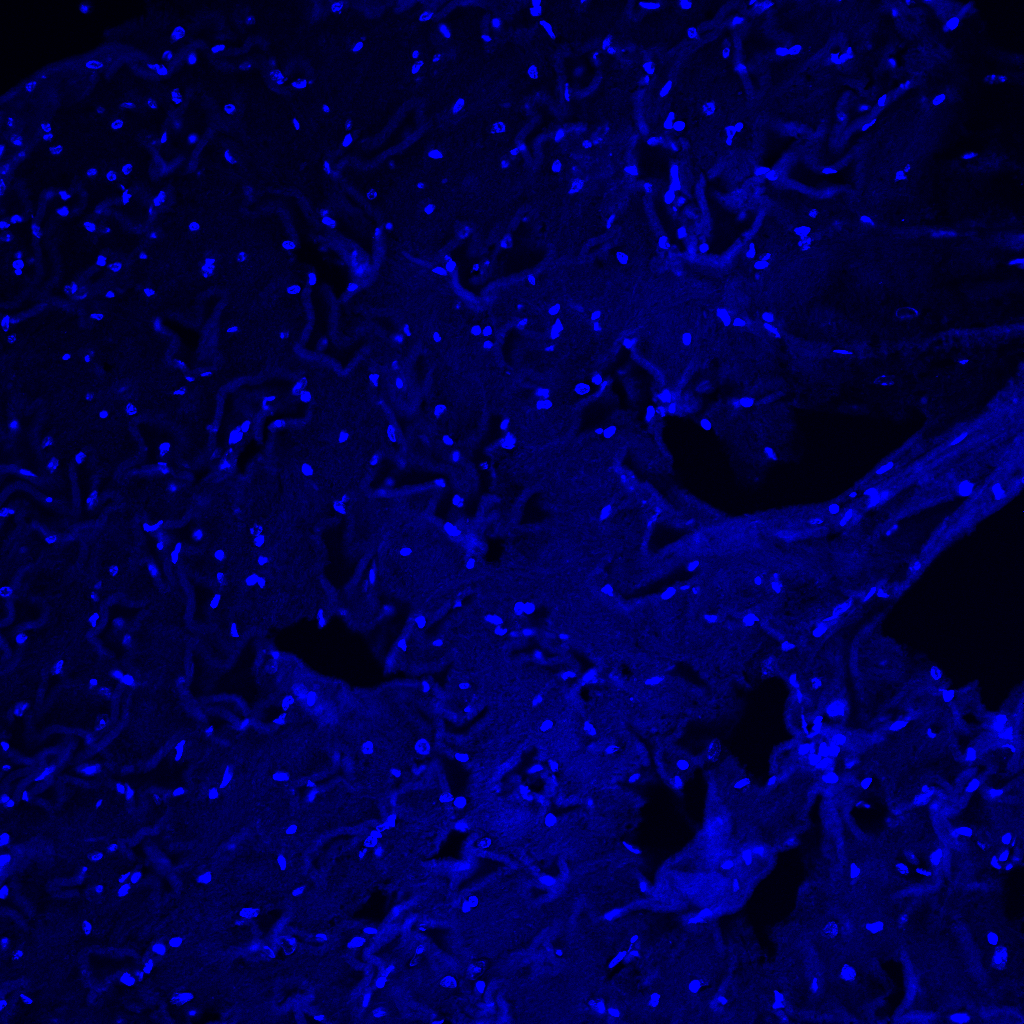

Supplement: Supplementary file 2 — Supporting Information [file ADVS-10-2300679-s001.zip › TMZ_Lipo_RGB_DAPI.tif]

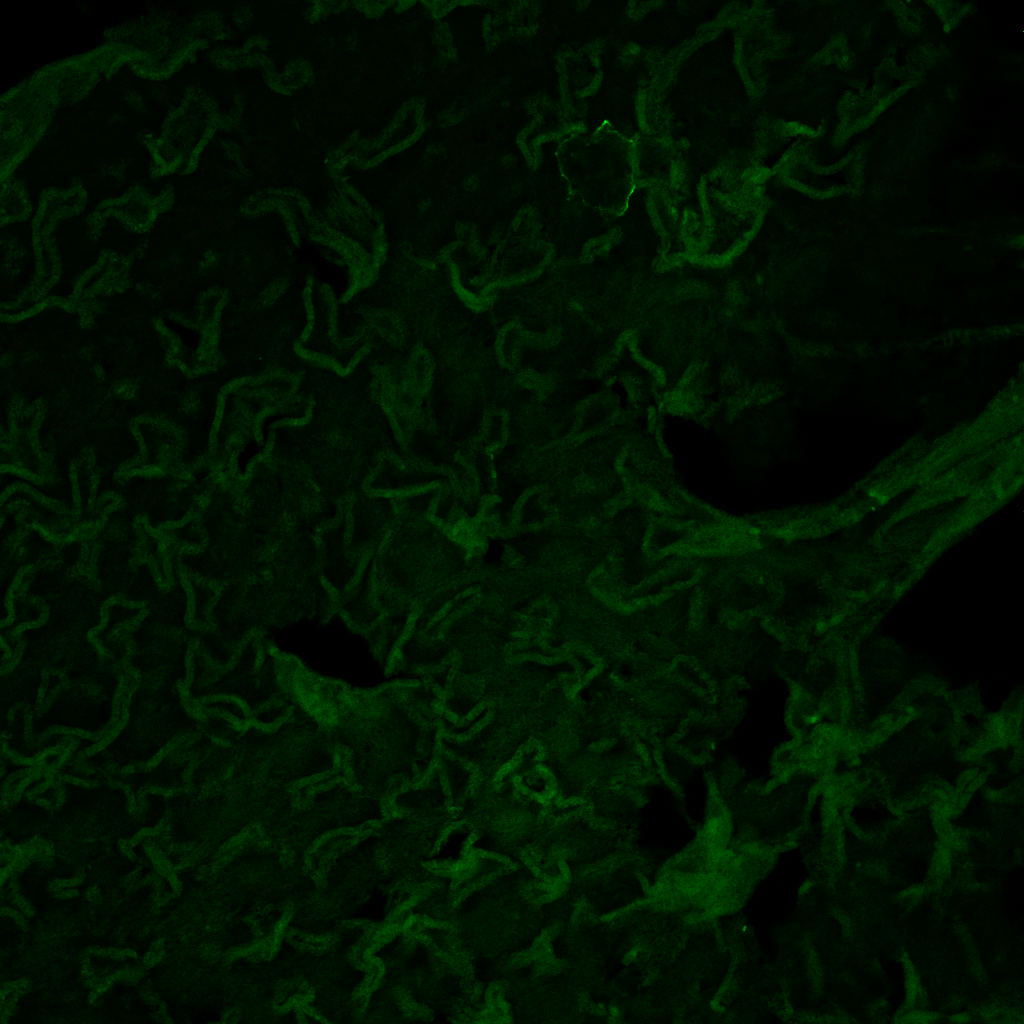

Supplement: Supplementary file 2 — Supporting Information [file ADVS-10-2300679-s001.zip › TMZ_Lipo_RGB_FITC.tif]
